# Miner Artist/Minor Artist? Class, Politics, and the Post-industrial Consumption of Mining Art

**DOI:** 10.3389/fsoc.2020.00062

**Published:** 2020-10-02

**Authors:** Jean Spence

**Affiliations:** East Durham Artists' Network, Seaham, United Kingdom

**Keywords:** mining art, Durham coal field, art market, ex-mining localities, nostalgia, regeneration

## Abstract

This article uses the recently discovered art work of a County Durham coal miner, Jimmy Kays (1886–1951) to highlight the terms in which coal mining art has achieved popularity and value in the post-mining period. Kays' work is considered with reference to the presenting narrative that promotes and markets mining art not only in terms of its intrinsic artistic quality, but also as a desirable commodity which, as a legacy of the mining past, can contribute to the revival of post-mining places. Maximizing the value that can accrue from mining art in post-industrial conditions involves appealing to the interest of the largest possible audience. The consequence of this is the dominance of a particular interpretation of the mining past. The art of Jimmy Kays does not conform to the conditions of the market, and cannot achieve a similar status. Despite its artistic qualities and its uniqueness as the product of a Durham working miner in the early twentieth century, it sits outside the dominant lexicon of coal mining art. The outsider status of Jimmy Kays is an example of a wider set of issues relating to the invisibility of working class creativity and the difficulties of achieving excellence or public acknowledgment in conditions that lack organizational support and in which value is established elsewhere. I argue that an understanding of the invisibility of art work such as that produced by Kays illuminates the exercise of class-based power in terms of the production, consumption, and range of meaning inscribed within popular mining art. Mining art that has been allocated value is in danger of being appropriated in ways that pacify rather than energize audiences, by foregrounding particular aspects of the mining past for purposes of consumption whilst submerging the issues that link more troubled aspects of the past with the present.

## Introduction

Since the demise of deep coal mining in the UK in the years that followed the 1985 defeat of the year-long miners' strike, public interest in “mining art” has grown. Artists such as George Bissill (1896–1973) and Gilbert Daykin (1886–1939) who depicted mining work and life from their own experiences have become well-known and sit alongside professionals such as Sir Frank Brangwyn (1867–1956) and H. A. Freeth (1912–1986) who represented mining life as observers (McManners and Wales, 2002).

In the northern coalfield, the work of the most popular miner artists came to prominence through the groups in which they developed their artistic skills. The paintings of miner Oliver Kilbourn (1904–1993) are particularly notable in the context of the Ashington Group (1934–1983) that met initially under the auspices of the Workers' Educational Association (WEA). The group was brought to public attention through Lee Hall's critically acclaimed play, *The Pitmen Painters*, and a collection of the paintings is now on permanent display in a specially designed facility at Woodhorn Mining Museum. Not all members of the Ashington group were miners, but the subject matter is predominantly of life in what was once understood as the world's largest coal mining village, built to serve five collieries, including Woodhorn (Feaver, 1988; Hall, 2008). Further south, the artistic development of Norman Cornish (1919–2014) and Tom McGuinness (1926–2006), two prominent artists of the Durham coalfield, was encouraged by their membership of the sketching group, established through a conscious effort to develop the arts, including literature and drama, within the Spennymoor Settlement (1931–1954) (McManners and Wales, 2006, 2008). As in Ashington, members, including non-miners, flourished through encouragement to paint what they knew from their own lives in a mining town.

The paintings of McGuinness and Cornish are central to the Gemini Collection of mining art which inspired the development of a Mining Art Gallery in Bishop Auckland. In preparation for the gallery opening in 2017, the collection was expanded by its owners, Bob McManners and Gillian Wales with a range of solicited donations. Amongst those are a set of cartoons, prints, and drawings by a County Durham miner, James (Jimmy) Kays (1886–1951) whose mining images belong to the first quarter of the twentieth century, predating most other items in the collection. A generation before the artists of Ashington and Spennymoor were representing their own lives, Kays was doing likewise, apparently without the benefit of tutor, organization, or support group to help him develop or exhibit his art.

Thwarted in his ambition to become a professional artist by family circumstances^1^, Kays' mining life began in 1899 and ended sometime in the 1920's. His reason for leaving the pit is unclear, but the 1920's was a decade of political turmoil in mining culminating in the 1926 General Strike and 7 month mining lockout against proposed mining wage cuts and extended hours. The defeat of the miners resulted in many miners, especially union activists, losing their jobs (Garside, 1971). Subsequently taking work as a dustbin man and later as a night watchman, Kays illustrated the local social life and characters in the mining village of Horden where he lived and where men in his family continued to work in the colliery until it closed in 1987.

**Figure 1:**
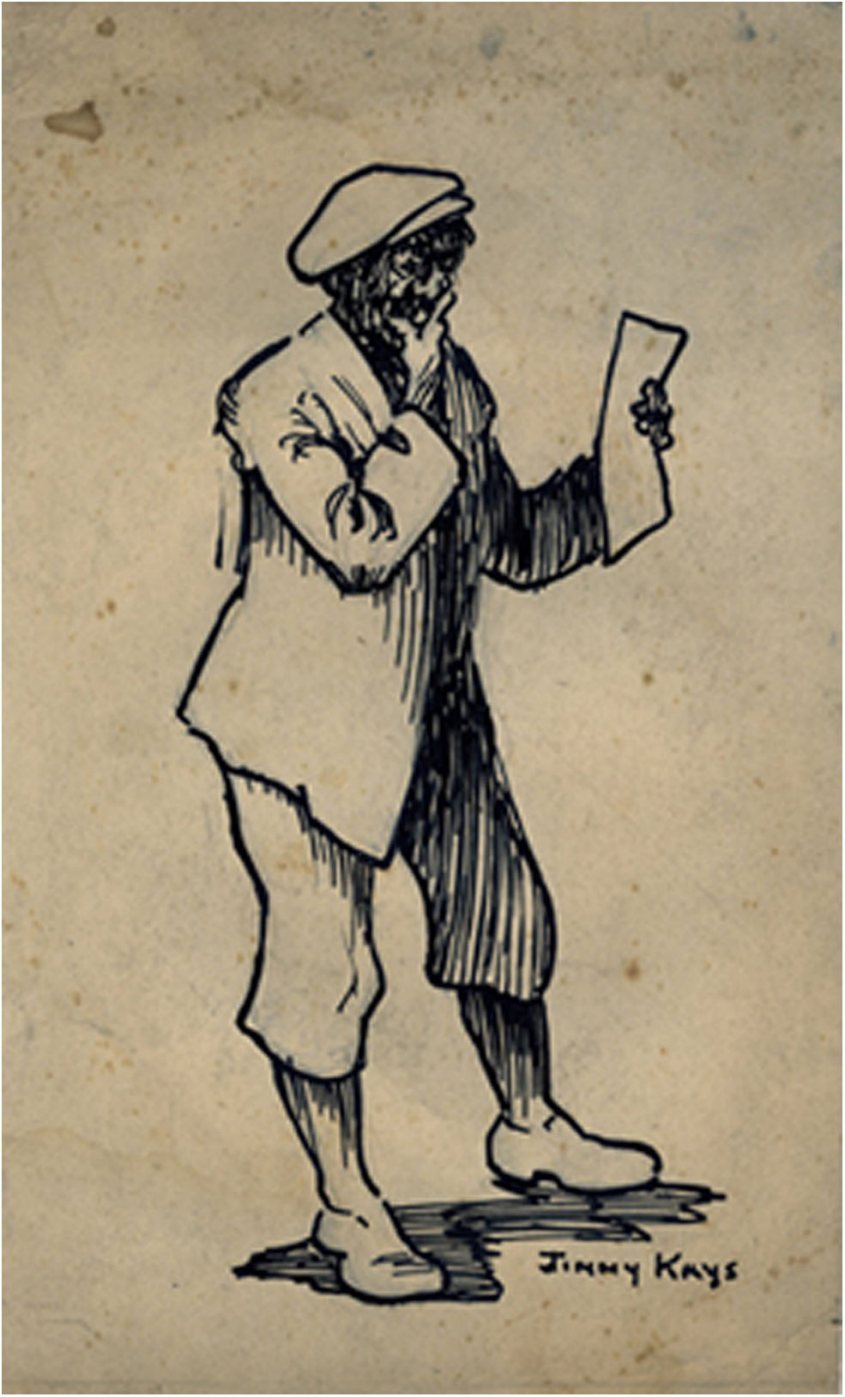
The offtakes question. *Author's collection*.

**Figure 2:**
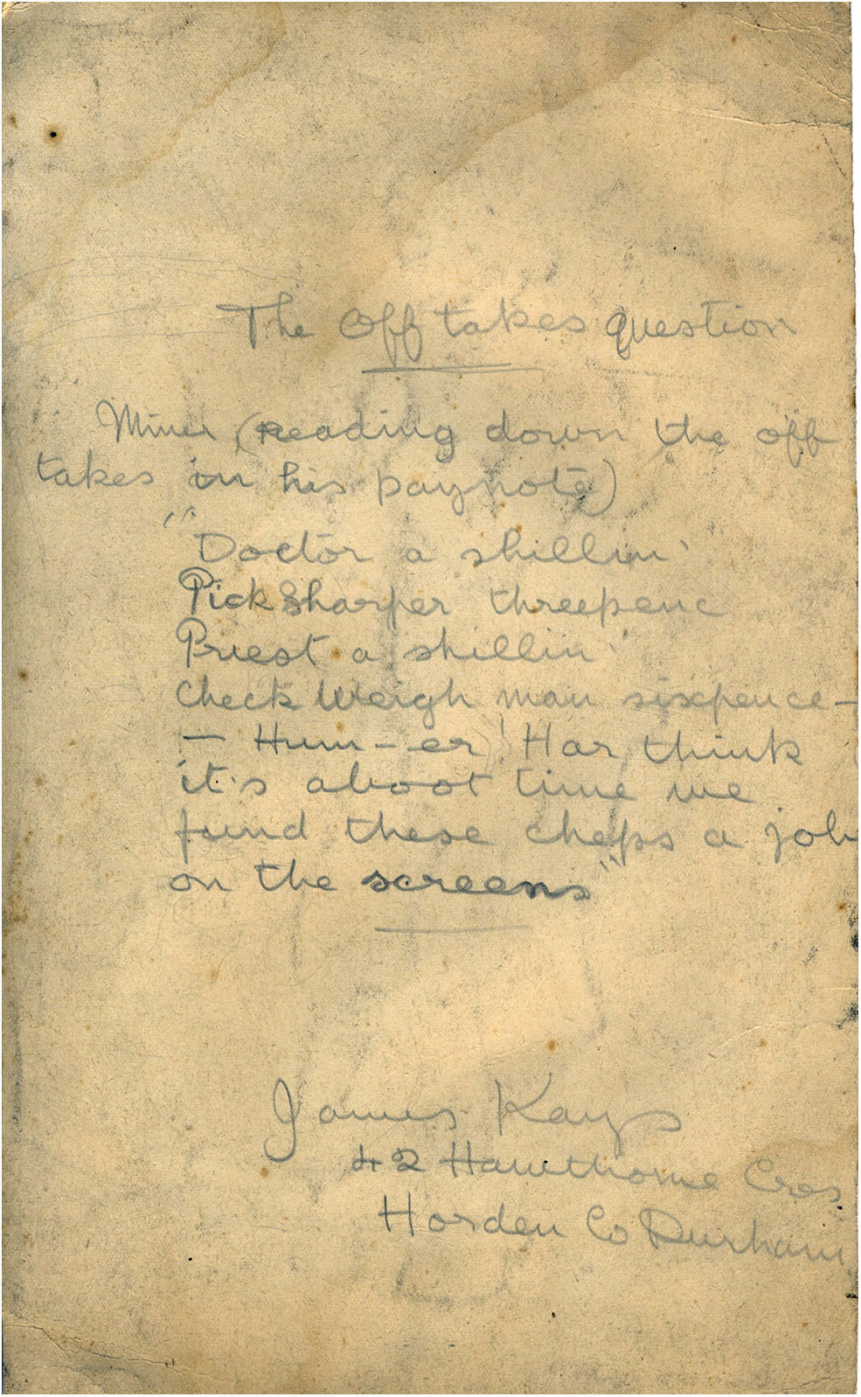
The offtakes question (reverse side). *Author's collection*.

**Figure 3:**
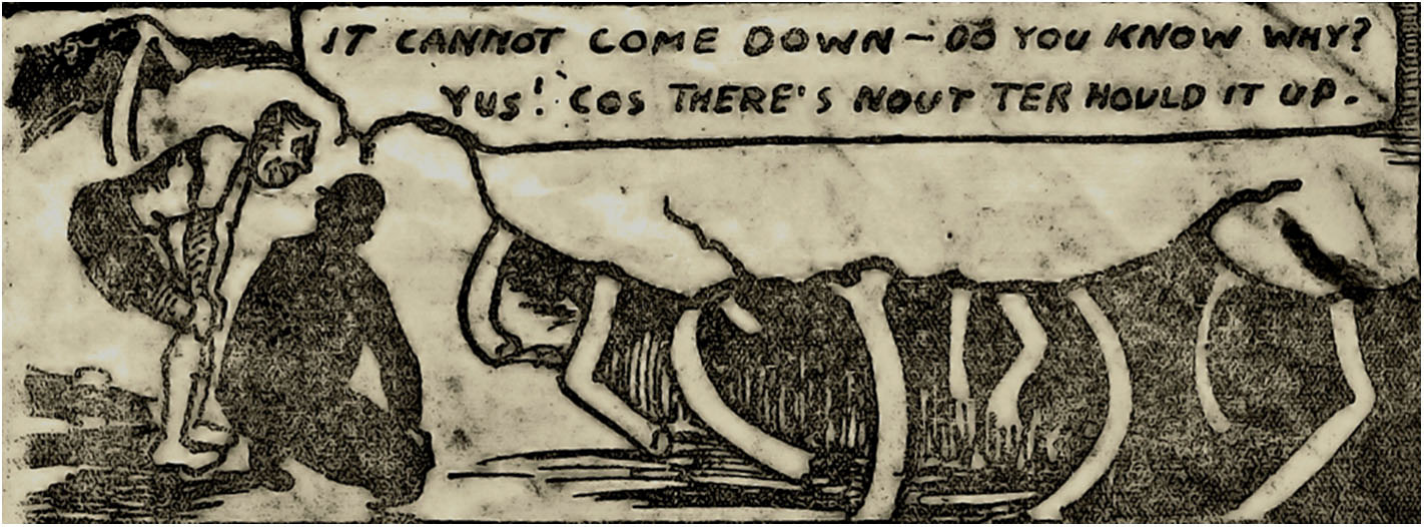
Nowt ter hould it up. *Author's collection*.

Kays' mining images do not fit smoothly with the “finished” paintings in the Gemini collection. He suffered from a lack of access to high quality art materials. The work is largely produced on cheap paper or card and comprises mainly cartoons, line drawings and lino prints, all of which require few materials, but none of which lend themselves to fully realized visual compositions. Nevertheless, the cartoons tell “stories” concerning his work and society, and offer a unique insight into Durham mining life in the early twentieth century. His observations of characters and relationships draw attention to many of the difficulties endured in mining life and work and he communicates these difficulties with a wry humor (Figures 1–3).

Unlike most of the miner artists in the Gemini collection, Kays is unknown. He produced cartoons during 1923 for a newspaper, the *Weekly Star*, that seems to have survived only for that year, but his work has otherwise remained hidden in his family. The death of a daughter who was not in touch with other family members brought some of the work onto the open market and l bid for it on eBay in late 2014. The auction starting price had been £10.00. My winning bid of £62.00 bought me thirty four items including original work and *Weekly Star* cuttings.

Under the protection of the Mining Art Gallery, the set from e-Bay will now be preserved in archive conditions and at some time might receive remedial attention with a view to showing it in the gallery. At present, there are no examples of Kays' work in the permanent exhibition nor has there been any public reference to these images preserved in the archive. In response to my question about the omission of Kays at the opening of the gallery, I was informally told that his art “did not fit the narrative” which gave cohesion to the permanent exhibition. I accepted this explanation but it prompted me to reflect upon “the narrative” in terms of what it might be and how it was being read and consumed in post-industrial conditions. This linked with a wider set of questions about mining and creative practice that arose from previous research and analysis concerning mining life and culture, and my current participation in a voluntary arts project in the ex-mining area of East Durham.

## The Research Context

The issues raised in this article arose from personal-political circumstances and activism rather than from the conventions involved in pursuing an empirical research project. Having been brought up in a Durham mining family in the mining town of Seaham, and being involved in women's activism against planned mine closures in 1992–1993, I began to write reflexively about the subject of identity, gender and mining politics as a sociologist and lecturer in community and youth work (Spence, 1996). Insights from this work were subsequently extended in partnership with Carol Stephenson to cover questions of community, activism, and cultural production specifically relating to gender and the narratives that articulate mining life with class politics in the UK (Spence and Stephenson, 2007, 2009, 2012).

Research undertaken with Stephenson included a review and analysis of poetry written and published by women during the 1984–1985 UK Miners' Strike and also a consideration of the significance of a series of photographs of miners taken and used by women involved in a campaign associated with pit closures in Nova Scotia, Canada. The evidence suggests a wealth of hidden creative and artistic skill and production in mining life. Much of this is revealed in conditions of political activism associated with mining that facilitates a brief public “flowering” after which most participants retreat (Williams, 2009; Popple and MacDonald, 2012). Although images, publications, and other ephemera survive the political conflicts that inspire them, subsequent public attention is mostly given to material produced by professional artists and commentators—including participant observers of the activism. The distinction between professionals and amateurs in the public allocation of worth implies that much of the artistic talent and creative output of working miners and their families has been unacknowledged in mining history. This raises questions about the forces that enabled some miner artists to overcome barriers, develop their creative skill, and achieve public recognition—and indeed my own role in bringing Jimmy Kays to public attention. Such questions articulate with issues of class, power, and structural inequality that informed my professional practice as a community and youth work practitioner and lecturer.

After retiring in 2010, I became more consistently involved in a voluntary arts organization, East Durham Artists' Network, (edan) which is committed to encouraging art and cultural practices in East Durham, and runs a small art gallery, the Art Block, in Seaham. The Art Block is the only dedicated art gallery in the East Durham coalfield, and to my knowledge there has never been another. The nearest public galleries are Hartlepool to the South and Sunderland to the north. Edan is largely self-funded through membership fees. The gallery, previously a public toilet, is leased free of charge from Durham County Council and occasionally small grants are accessed from various sources to enable specific projects to proceed. Although not organized to promote “mining art,” edan is responsive to the legacy of mining. The east Durham landscape still carries the scars of coal, mining is a continuing reference point for local identities, and the history of mining is one strand in efforts to reinvent East Durham for leisure and tourism. Thus, member artists, including ex-miners and artists from mining families, have created publications and organized exhibitions and activities that focus upon mining themes. The local response to such activities in terms of numbers of visitors to the gallery and the level of participation of those visitors greatly exceeds those of other themes. A Jimmy Kays exhibition attracted over 500 visitors between 16th January and 25th February 2016—about four times the average for a winter month normally very low on footfall. Edan's activities do not include “research.” To undertake formally constructed research in this setting would run counter to my role as a volunteer and be problematic in terms of the standing of the organization in the locality. Nevertheless, producing art work related to mining, helping to curate exhibitions that represent mining, participating in associated art workshops, and being present in the gallery, provide opportunities to hear unguarded opinions and to engage in reflective conversation about art and mining which would not be otherwise possible. The experience has suggested that whilst there are high levels of interest in the visual representation of mining life, there are low levels of confidence about “art knowledge” and artistic skill in east Durham.

I chanced upon the Jimmy Kays images on eBay during the lead-up to an exhibition at the Art Block arranged to commemorate the thirtieth anniversary of the ending of the 1984–1985 miners' strike. The selection eventually displayed in that exhibition attracted enthusiastic attention and commentary from visitors. Later, thanks to support from Durham Heritage Coast^2^, it was possible to print and frame copies of the originals for further local exhibitions. The inaugural exhibition, at the opening of a new Heritage Center in what was previously a mine ambulance station in Kays' home town of Horden, generated a great deal of media excitement and received wide publicity as the “discovery of a miner artist” (e.g., Engelbrecht, 2015; Prince, 2015). However, local visitors to the Kays exhibitions spoke about the images not so much as art, but in terms of personal mining identities and histories. They also frequently referred to their own (thwarted) interest in artistic practice.

The eBay collection revealed little about the artist other than his name, address in a council house in Horden, a date of 1912 on one drawing, and some *Weekly Star* cuttings dated 1923. I therefore embarked on a research quest firstly to locate surviving members of the Kays family, and secondly to unearth any surviving documentary evidence of his life. Family members were contacted thanks to an article in the *Sunderland Echo* (Stoner, 2015). His descendants offered information about Kays' life and personality and also revealed a further three collections of his art, plus one or two individual pieces in their private ownership. Otherwise, little documentary evidence and few photographs survive. The census, war records, newspaper reports, and mining archives provided scant information. Official documents also led to confusion between Kays the artist and a cousin with the same name. Research was further complicated by the fact that Kays was born to a single mother and his father is not named on his birth certificate. Later, he took the name of his stepfather, Penman, and then on marriage, returned to his given name. Eventually I was able to reconstruct something of a Kays biography and produced a short pamphlet about the collection (Spence, 2015). The documentary information revealed nothing about Kays the artist. The only sources for such information are the memories of his last surviving son, who was 11 years old when his father died, the clues present in the work itself, and the few *Weekly Star* cuttings of cartoons attributed to “Jimmy Kays, the Horden Miner.” The difficulties involved in “finding” Kays raise a volley of questions about the invisibility of working class lives and the lack of value accorded to the fruits of working class creative labor, of which the known miner artists appear to be an exception.

The following discussion draws upon my own responses to Kays' images, to the mining-related art that I have encountered via the work of edan, and in visits to mining art exhibitions. I have appreciated, enjoyed, and identified with mining art, but my response often includes a sense of “lack.” Sometimes this is associated with the subjectivity of gender and the exclusion of women from mining and mining art, but sometimes it derives from my experiential awareness of the complex social relationships in mining which continue to impact on the post-mining experience but are mostly absent in mining art. In this regard, insights gained from participation in workshops in the Art Block using the concept of Social Haunting have been helpful (Gordon, 2008). The workshops, (“ghost labs”), conducted as part of an Arts and Humanities Research Council research project were intended to highlight the potential of creative expression in addressing troubled and repressed histories, in this case specifically that of mining and the miners' strike (Munday, 2017; Spence, 2019). They provoked reflection and analysis that fed into this article regarding the role of mining art in energizing local people toward artistic and cultural production in contemporary post-mining conditions.

## Mining Art and the Death of Mining

Mining art has come to prominence in the UK in the context of the demise of an industry that had been synonymous with British wealth and global power. Deep mining was declining before the final closure programme of the late twentieth century, but coal had remained a natural asset and mining was heavily subsidized by the government. From the late 1950's, a programme of restructuring and closure had been carefully managed by the National Coal Board (NCB), responsible for the nationalized industry, in collaboration with the National Union of Mineworkers (NUM) (Hall, 1981). As late as 1980, the NCB Chairman could write in a catalog accompanying a national exhibition of mining art:

*Coal has played a vital part in the British economy for more than three centuries and was the foundation of the industrial revolution that made Britain “the workshop of the world.” Today the coal industry has an assured future, with several hundred years of reserves still available, and will continue to play that central role* (Siddall, 1980).

One year later, the Conservative Government under Margaret Thatcher, elected in 1979 in a climate of hostility toward trades unions, was threatening that “assured future,” proposing to close 23 pits. Faced with NUM strike threats, the government temporarily retreated. The decisive Conservative re-election in 1983 was to radically change conditions for managing the coal industry. Government determination to reduce state subsidies and accelerate pit closures provoked a bitter strike beginning March 1984. In March 1985, the NUM admitted defeat. The consequence was the destruction of the power of the NUM, demoralization amongst the miners, and a rapid decline of the industry (Goodman, 1985; Williams, 2009, 2019).

The 1984–1985 strike enacted the collectivist values of mining life, and its defeat precipitated not only the end of mining, but also the denigration of those values. The NUM had organized successful strikes in 1972 and 1974, resulting in the fall of a Conservative Government. Both sides of the conflict understood mining to be an exemplar of trade union, and indeed, working class collective influence (Hall, 1981). Destroying the NUM, led by its avowedly socialist president Arthur Scargill, was pivotal to the government's wider determination to eradicate socialism from industrial organization in the UK (Goodman, 1985). Defeating the miners was both a means to implementing pit closures, and also to weakening the whole British labor movement, enforcing an historic shift in classed social and political values as well as industrial relations. The subsequent privatization of the coal industry in 1994, and the radical closure programme that ended deep mining in the UK has left a legacy of deprivation, social dislocation, and bitterness in ex-mining areas, and a level of resentment in the wider labor movement that remains unresolved today (Beatty et al., 2019; Williams, 2019). Such conditions shape the post-industrial reception, deployment, and consumption of the remnants of mining, including its art and culture.

Just as coal mining had been at the core of the industrial revolution, its deconstruction was pivotal to the de-industrializing revolution that involved streamlining the economy to compete in the “free” global market and privatizing state-run industry and services. Neo-liberal Conservativism favored the service and creative sectors over manufacturing, promoting enterprising individualism at the expense of collective organization and social welfare. Such processes stimulated the art market and the growth in art collecting as an opportunity for private investment. Mining art, as a specialist interest, and one connected with an industry that was rapidly disappearing, gained enormous financial value in this atmosphere.

Independently, McManners and Wales began collecting mining art in the early 1970's (McManners and Wales, 2006). At that time, the subject matter was unfashionable and the miner artists, as working class “amateurs,” lacked status. Professor Jean Brown, Director of Northumbria University Gallery, has suggested for example, that because Cornish was a miner without professional art training, his paintings were stigmatized in the “fickle” art market and that even today he remains underrated as an artist in these terms (Horton, 2019). Yet the post-mining rise in value has been exponential. Mining solicitor, Iven Geffen paid under £10 for paintings by Cornish and McGuinness in the 1950's that sold for thousands at auction in 2015 (Henderson, 2015). The death of an artist further affects the price. Cornish prices rose sharply after his death in 2014 and have benefited from promotional activities marking the centenary of his birth in 1919. His oil painting of the Acadia Cinema, Spennymoor, is currently advertised for sale for £26,000 (Castlegate House, 2019) whilst prices on the official web site range from £2,500 to £13,950 (Norman Cornish Ltd., 2019). Cornish was prolific, painting throughout his long life; it is almost impossible to find for sale work by other highly rated mining artists including McGuinness who died in 2006.

Mining art expresses and validates memories of a previous era which has left traces in the physical qualities of once lively but now depleted places. As such, death increases not only its financial but also its emotional value. Mining art can contribute to the mourning process (Roberts, 2007). However, financial and emotional values diverge. People who suffered most directly from mine closures are those most likely to find mining art prices prohibitive. Conversely, emotions hardly count in investment decisions, even if they shape the interest. The art that emerged from mining places is beyond the purchasing power of most people who live in the shadow of the mining past. The average income in Spennymoor is <£25.000 (Spennymoor AAP, 2017). In Ashington it is <£23,000. Meanwhile in Sunderland, a town that suffered particularly from de-industrialization and whose colliery closed in 1993, it is <£15,000 (Dowson, 2019). A recent report suggests that if grouped as a region “the statistics would probably show the former coalfields to be the most deprived region in the UK” (Beatty et al., 2019, p. 44). Ironically, the very processes that have added value to mining art are those responsible for the collapse of the places that conceived and nourished the talent of miner artists. Thus, the art is distanced from its source in working class life. It now belongs “elsewhere”—not only in the past, but in terms of ownership and control.

Efforts to make mining art publicly accessible through museum and gallery exhibitions and their derivative consumer goods, are responsive to its emotional value in ex-mining localities. However, in order to be sustainable, the organizations concerned must both take advantage of opportunities offered in the post-industrial economy, and appeal to the personal interest of a much broader constituency than those directly affected by mine closures (Scott, 2009). This is possible because of the importance of mining to British industrial history and because so many families have memories of mining and continuing links with ex-mining places.

## Mining Art, Memory and Nostalgia

The importance of coal mining to British collective memory can hardly be overestimated. From the start of industrialization, coal powered British manufacturing and imperial expansion, sustained its wars, and warmed its homes. At its height in 1920 when Jimmy Kays was mining, it employed 1,191,000 men. As British global power waned, coal mining contracted unevenly but it remained significant, employing 237,000 workers in 1980. By 1984, only 180,000 workers remained, but the ensuing strike energized a field of supporters who identified with mining life and values, projecting mining to the center of British political consciousness and setting the terms for alignments that remain influential today. In the 15 years following the strike, the coal industry lost 169,000 workers. When the last deep mine, Kellingly closed in 2015, 1,000 jobs were lost (Sönnichsen, 2020). It is hardly surprising that coal mining and its fate continues to have emotional resonance and even to “haunt” large numbers of people. However, that haunting is different in quality for men and women who have different relationships to and memories of mining and its politics. The inheritance of mining is a complex matter.

The death of its industry means that mining art now forever represents a world that is past and indeed “lost”—but lost in many different ways. For those who encounter mining images from the perspective of their interest in mining rather than art, engagement is first and foremost with the past and with the meaning of that past in contemporary lives. Encounters with mining art are not direct. They are filtered through organizations such as galleries and museums, inflected by the subject matter of the images and the decisions of curators. Ensuring that exhibitions are financially viable demands that audiences become consumers. Attracting the widest consuming audience is achieved largely by obscuring differences and appealing to a shared mining heritage. The lost world that is integral to the history of the nation, is something that we can all own and, whatever the different qualities of our loss, in viewing the art of coal we can all share in the bittersweet nostalgic pleasures that remind us of where we came from.

Significantly for the fate of the Jimmy Kays collection, his images are very specific in time and place. His cartoons in particular, with their accompanying captions in “pitmatic” dialect^3^, are situated very firmly in Durham (Griffiths, 1994). Meanwhile, the work predates the time frame that activates living memory which is an important characteristic of the nostalgic qualities of so much mining art. Kays' work was produced before the circumstances that encouraged the development of most of the miner artists from the 1930's onwards.

Following the 1926 defeat, miners' politics and union activism were in abeyance. The depression and mass unemployment of the 1930's encouraged voluntary organizations to help in the “distressed areas” (Hannington, 1937; Williamson, 1982). The Ashington WEA class and the Spennymoor Settlement were both middle class socialist and left-liberal responses to the difficulties endured by impoverished mining communities. The mining art nurtured through cross-class institutional contacts in the 1930's, became more publicly visible because of the Second World War. Mining became once again a key industry and men were conscripted from across the UK to produce coal. The conscripts (Bevin Boys) included some, such as Ted Holloway, who came to be recognized amongst the best miner artists (McManners and Wales, 2002). Meanwhile Graham Sutherland and Henry Moore (a coal-miner's son) were employed by the government to illustrate the contribution of mining to the war effort, and mining became an acceptable artistic subject. After the war, mining was accepted as essential to rebuilding the economy. Nationalization in 1947 offered the hope of calmer industrial relations and the NCB, facing manpower shortages, was keen to foster good will and recruit new workers (Illingworth, 1952). Its efforts to enhance the image of the industry included promotional film, photography, and painting^4^. Professional artist H. A. Freeth was employed to visit mines and produce a series of portraits of miners that were published in the NCB newspaper *Coal: The Magazine of the Mining Industr*y (e.g., Freeth, 1951). Meanwhile, its welfare arm, the Coal Industry Social Welfare Organization (CISWO), sought to stimulate positive leisure activity in the coalfield. The annual art competitions it organized encouraged the talents of artistically inclined miners, and offered opportunities for their work to be publicly displayed.

**Figure 4:**
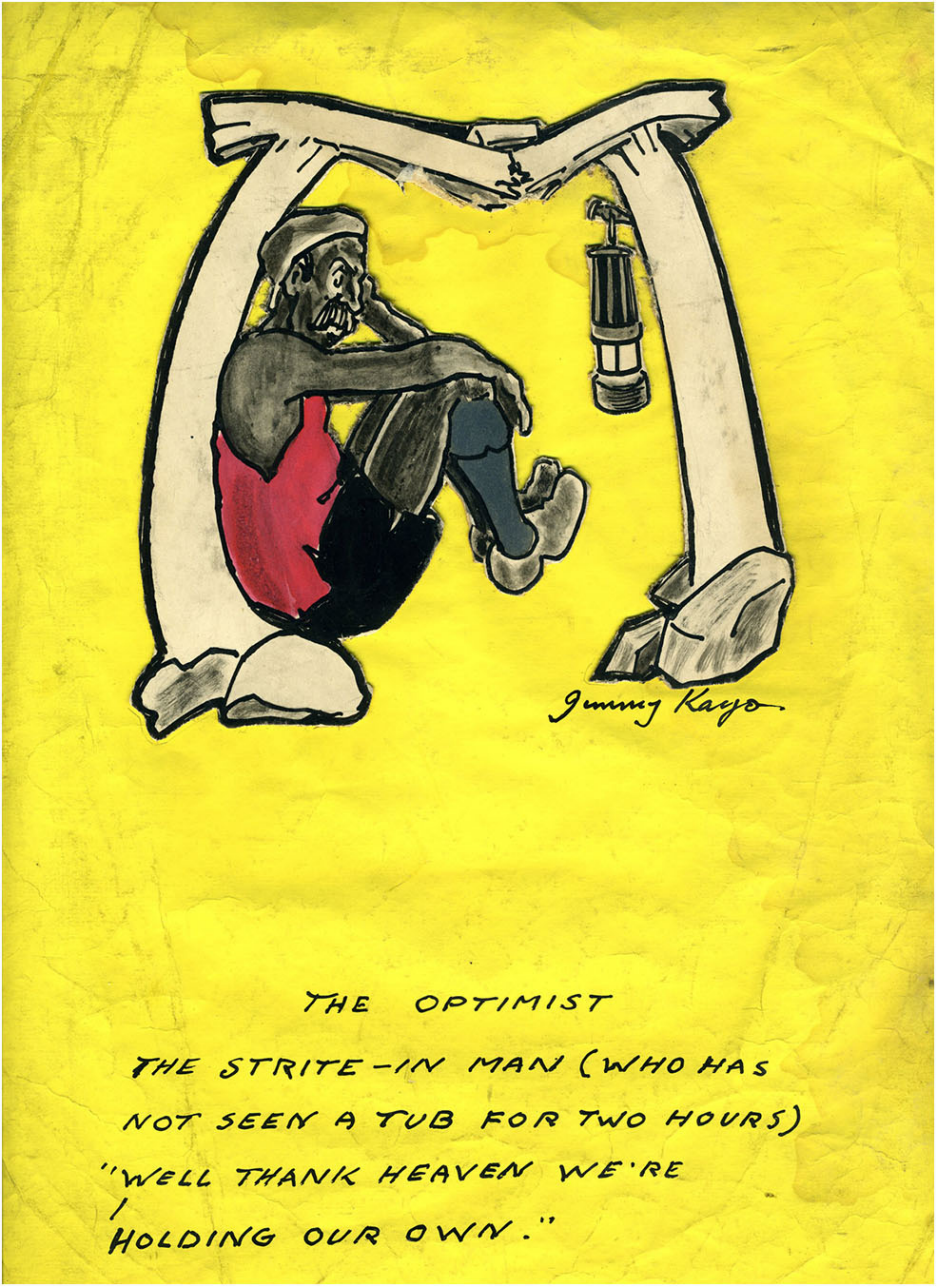
The optimist. *Author's collection*.

It is this period of working class validation, underpinned by new securities offered by the emergent welfare state that underpins many memories of the mining past. Much mining art reflecting mining life at that time provokes simple nostalgic responses, but that is offset by other images relating to the actual work of mining that reminds the viewer of the dangers and difficulties of mining. Many miners disliked their work and the post-war period that nourished mining art, also nourished dreams and opportunities that involved escape. The securities of that time offered a bedrock for building alternative futures—including for those artists who left mining in order to paint it. Thus, the nostalgic responses to mining art can be as much about the sense of security and hope for a different future inscribed in post-war mining images as about the mining itself. That sense of security and hope is absent from Kays' work. Although many of his images are humorous, it is a humor derived of resignation to insecurity. The cartoons foreground powerlessness in a world in which escape was hardly a possibility (Figure 4). In this fundamental sense, Kays' work does not fit one narrative that makes mining art attractive in post-industrial conditions.

The end of mining coincided with the end of previous securities. The imposition of neo-liberal policies radically altered employment and other institutional relationships, creating work-based stress even amongst the once-elite skilled and professional classes (Young, 2007; Baccaro and Howell, 2011). In the vertiginous maelstrom of post-industrialization, the nostalgic appeal of security is powerful and can sanitize the painful features of the remembered industrial past.

Strangleman (2013) has argued that there are “radical or oppositional” aspects to nostalgia, which can provoke critical questioning of the present. Opportunities for the stimulation of such nostalgic responses undoubtedly exist in coal mining art—perhaps most notably in the work of McGuinness (McManners and Wales, 2006). However, the type of nostalgia stimulated through the filters of the tourism and leisure markets which frame public access to mining art, is much more likely to be reflective in ways suggested by Boym (2001). Ambiguity between security and danger might provoke critical reflection but is more likely to result in passive engagement with a past that seems absolutely disconnected from the present. There is a tendency to romanticize mining in national mythology, and also in working class politics such as witnessed during the 1984–1985 strike (Samuel et al., 1986). Romanticism feeds directly into the consumption of mining as “heritage” in the museum and gallery contexts through which “obsession with the past reveals an abyss of forgetting and takes place in inverse proportion to its actual preservation” (Boym, 2015, p. 13).

The descriptive and figurative images of mining life that are a significant part of the oeuvre of mining art exemplify past certainties in a volatile present, reminding viewers of a place where class and gender identities were given, social values clear, and families seemed cohesive. This obscures a world that Jimmy Kays shows, where repression, inequality, poverty, dissatisfaction, and dysfunctional families were as much a feature of everyday life as they are of any other social history. These features of mining did not disappear at nationalization but have been discarded in the collective discourse, driven into individual memory. Difficulties in mining society are not foregrounded in the images made by established miner artists for whom everyday scenes are largely benign. Before such images, consumers can collectively remember and identify, but any dislocation between personal memory of mining life and the dominant narratives in the art work is individualized. The art itself and the context in which it is consumed offer no resolution for this disconnect. We are not invited to engage painfully with the art on display in post-industrial contexts but rather to actively enjoy the stimulus to fond memories.

The tension between the securities of mining life and the insecurities of the actual work encourage a more resigned accommodation with the conditions of the present. Some mining art emphasizes masculine strength but the focus is predominantly upon the physical distortions and pain caused by mining labor. Kays' work also depicts the difficulties and dangers underground work but significantly, he is more concerned with working relationships and the unfairness endured by the system of allocating work which renders some individuals powerless. In this sense, Kays' images are a reminder of injustice in mining. Mining work was not only difficult, it was unfair. Such unfairness is not located only in the past and in mining but was always there and many of the gains won by workers through trade unions and collective action have now been lost. In this sense, Kays' simple cartoons are continuous with the present and more likely to provoke more oppositional aspects of nostalgia (Strangleman, 2013).

Images generated from mining life during that brief period between the second world war and the end of the 1950's, when the demand for coal began to shrink, implicitly affirm the virtues of social cohesion, cross-class collaboration, national unity, and indeed, national glory. The historic moment when the interests of miners and nation were as one, also nurtured non-mining aspirations and opportunities, many of which were realized. Today many families, across all classes–including the Duchess of Cambridge, who have no direct link with mining, can identify coal mining forebears. From a distance, the success of mining art in itself is an affirmation of the possibilities of social progress and mobility in which personal endeavor can prevail. The classed relations of power that survived nationalization and the social democratic consensus to eventually show their face in the conflict of the 1984–1985 strike and that continue to characterize the inequalities of post-industrial economic and social organization are not revealed in conventional mining art and therefore cannot disturb this view.

The broad appeal of “authentic” mining art lies at least partially in a conservative reading that emphasizes the role played by mining in the narrative of nation. The success of mining artists, the market value of their art, and their acceptance into the art establishment adds weight to this perspective. This is defied in the images and the fate of the work of Jimmy Kays. The conservative evaluation venerates a particular quality of art and idealizes a very particular moment of the past within a linear and progressive understanding of history. In the UK, the most prized mining art is by definition about the past, and its success implies that such a past always promised a brighter future. The 1984–1985 strike was in these terms, an aberration, a misguided, if understandable effort to reverse historic inevitability. Insofar as its appeal is to memory and to a simple or reflective nostalgia for what has been lost, most mining art, including that which references the union and the strike can be consumed without ambiguity by a wide range of people from across the political spectrum, whose mourning for a lost past is mitigated by the reassurance that they exist in that better future. Miners can be now comfortably claimed and acclaimed within the national story without irony (Horton, 2019). As Lee Hall describes the films sponsored by the NCB after nationalization: “Although they are clearly about mining, they are even more essentially about Britain” (Hall, 2009). The 1984–1985 strike, fought in terms of irreconcilable class-based values that precipitated the death of the industry, has been incorporated through a mythic history of national consensus. Disconnected from its source in working class life, mining art is subjected to an external gaze that creates a narrative that is partial. The work of Jimmy Kays, a minor working class artist, whose work speaks of poverty in its form and entrapment in its content, belies this narrative.

The disconnection from mining as a living occupation, and from the classed conditions that inspired the art, began in the lifetime of those miner artists who gained public recognition through organizational networks, sponsorship, and patronage associated with middle class interventions, art education, and the art market. Jimmy Kays' work stands outside such influences. He lacked cross–class or organizational connections. Living and working in what was a relatively new mining settlement of Horden, whose colliery opened in 1900, Kays seems not to have had access to voluntary community organizations whose support might have helped him develop his art and access external opportunities (Davis and Cousins, 1975). Except for the brief employment by the *Weekly Star*, he made art without external influence. Later miner artists who risked leaving mining to become full time artists became immediately subject to the buyers' market. The impact of this is evident for example, in the later work of Cornish and in some of his commissioned pieces^5^. Similarly, for those ex-miners, including Robert Olley and Bill Hindmarsh, separated from their source by the death of mining and forced to produce mining art from memory, the direct link between lived experience and their art is broken. For Jimmy Kays this did not happen. When he left mining in the mid 1920's, he stopped producing mining images, shifting his attention to everyday life, albeit in a mining place. His work is entirely of his present. In contrast, the post-industrial circumstances in which mining art flourishes encourages both artist and audience to focus upon the past without raising discomforting questions about the present. Thus, a gap has emerged between the contemporary lived experiences and remembered histories of struggle in ex-mining populations, and the lost and retrospective world that serves consumers.

Coal mining shaped the economy, social structure and local culture of all who lived and worked in mining districts for two centuries. The late twentieth century mine closures involved a systematic assault on traditional working class employment, organization and culture. For mining localities, patterns of work, social, and personal relationships were forcefully reshaped in ways that defied the expectations inherited from of the past so absolutely, that the past became the only “known” fixed point of stability. When the present is degraded and the future cannot be imagined, the past can become a place of retreat and nostalgia (Boym, 2001). Recognizable images of that past can validate received identities and steady instabilities associated with change. Mining art itself is part of what has been lost but as a visible residue, evidence of “what we were,” it is viewed with reverence, respect, and longing. Thus, the oeuvre of mining art is an important reference point for living artists who are ex-miners and for an audience of ex-mining people. It does not in itself offer resolution for the trauma of imposed loss, but ownership of a proud past can be encouraged through the public recognition of its art. In these terms, whether produced in the present or the past, art that uses the mining past as its subject becomes an object of desire to be consumed in a regenerated and re-formed economy that uses nostalgia to promote and market “heritage.” The appropriation of the mining past in these terms reinforces mining connections and identities but deprives mining history of its complex substance and contradictions. Art that was inspired by the experience of mining does not throw light on that contested class history in which miners and mining culture have been more denigrated than admired, and against which they struggled to improve their conditions of existence not only through industrial conflict, but also through industrial, social and political organization, the substance of which died with the industry.

## Degeneration and Regeneration

The earliest successes of miner artists depended upon the patronage of middle class social activists who shared their skills and social contacts (Gilchrist and Jeffs, 2001). Later, the NCB and adult education classes fulfilled a similar function. Would-be artists in contemporary post-mining places have access to informal educational and recreational opportunities, but mainly in organizations which are concerned with helping post-mining populations to participate constructively in post-industrial renewal. Opportunities offered in such circumstances are circumscribed by aims and objectives set elsewhere and in terms not relevant to art itself. In today's market dominated climate, voluntary and adult educational organizations, including the WEA and the remaining settlements, survive mainly through indirect deference to the policy demands of the central state. Their funding depends upon clearly defined objectives that are “delivered” by professional community and educational workers for whom politically referenced activity can be problematic. Significantly, the ex-coalfield areas of England have attracted little direct funding from the Arts Council (Hansard, 2018). The *Creative People and Places* initiative within the Arts Council that “focuses on parts of the country where involvement in arts and culture is significantly below the national average,” includes ex-mining areas such as East Durham (Arts Council, 2017)^6^. Post-mining arts and culture have been supported by Government and by charitable trusts mainly through the lens of regeneration. As such, the goals are about adaptation. These processes might encourage creative 'skill' but they do not encourage critical engagement with post-mining life. Encouraging local participation to encourage active citizenship is often an underlying objective of community based initiatives. Making art is one means to that end.

**Figure 5:**
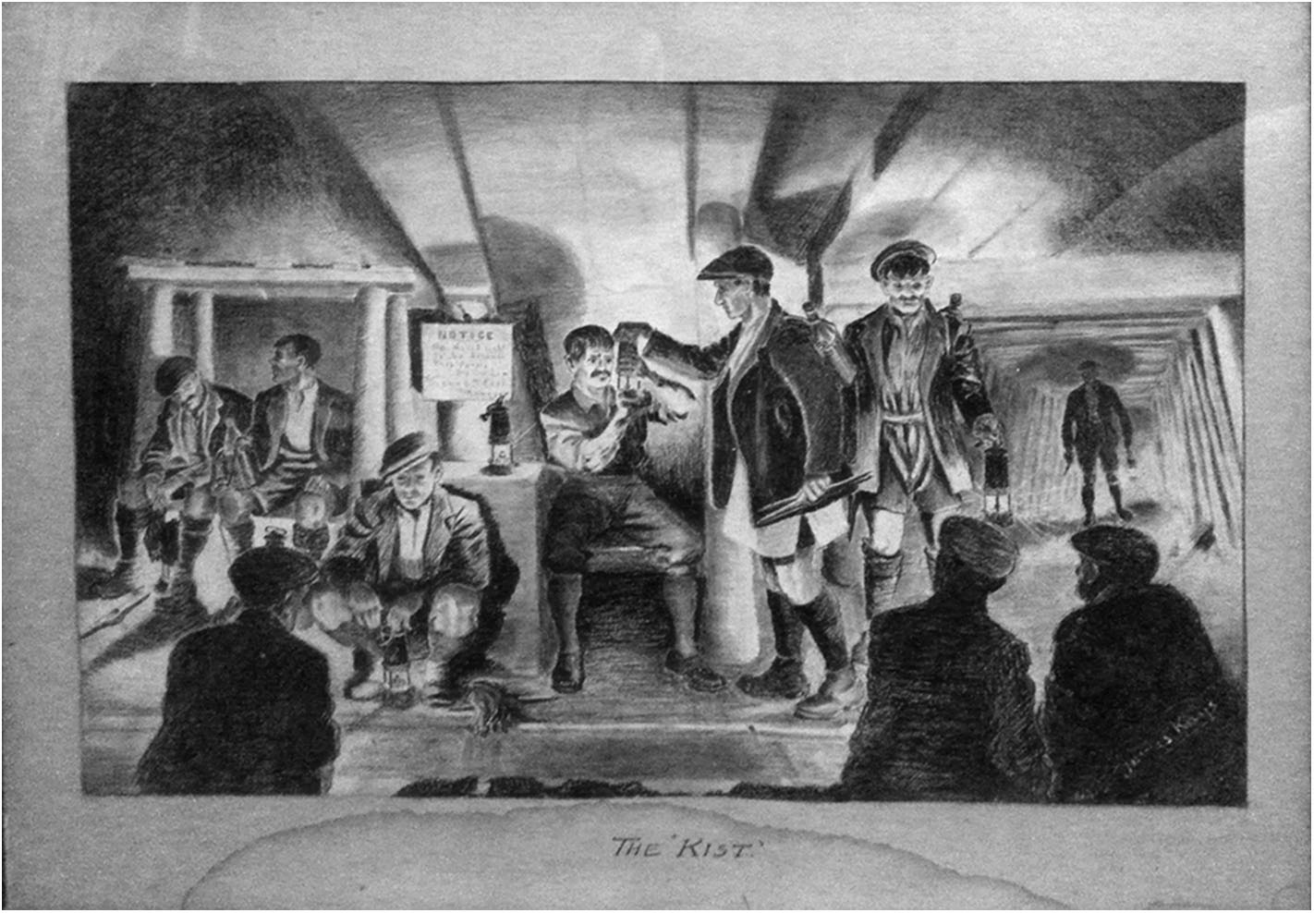
The Kist. *Private collection, W. Kays*.

The experience of the 1984–1985 miners' strike and of pit closures seriously dented trust in the value of civic and industrial organization among ex-mining populations. The defeat of the strike and the inability of the miners' union and its welfare and recreational organizations to prevail against the tide of neo-liberalism implied a loss of hope, energy, and confidence in the subjective power to influence the present, to escape from it, or to imagine a better future (Bennet et al., 2000; Perchard, 2013). This too limits the prospects for artistic expression that references the present condition of post-mining life or that facilitates building on the work of miner artists in ways that are not simply derivative or retrospective. The drive toward transition after mine closures included the hasty demolition of mining structures that were closely aligned with working class industrial politics. Such loss included demolition of the “Big Club,” the Working Men's Club in Horden where a Jimmy Kays composition “The Kist,” having been donated by one of Jimmy Kays' sons, had been on display in the Committee Room for many years (Figure 5). The picture, the only one to have inhabited a (semi) public space, was returned to the family, to be hidden once again from public view. The fate of the club and “The Kist,”^7^ which his family consider his “best work,” produced probably in 1919 as a present for his in-laws after the death of his first wife, could be a metaphor for the fate of those whose identities, knowledge, and skills were displaced by the death of the industry.

The end of mining set a seal on the past, domesticating it and obliterating its visible remains whilst introducing new public constructs for the future. Changing physical and cultural landscapes reflected the now uncharted landscapes of people's lives and their lack of control over how those landscapes would be reshaped and remembered (Atkinson, 1999; Ling et al., 2007). Whilst the state and private interests engaged in a highly politicized and exploitative feast of “regeneration,” the institutional and cultural life of ex-mining localities was destroyed or repressed. In compensation, ex-miners and their descendants were presented with a pastiche of their past through a process of memorializing that replaced mining with its representation as heritage whilst enabling no place for authentic representation of the present or the critical interrogation of the past (Griffin, 2006). What was on offer was a mediated and reframed perception of mining art and culture that would sit in the landscape as in a cemetery, whilst the body that had served the mines decomposed.

In post-mining environments, public space is degraded and contested before it is regraded. The will to remember mining in the regraded landscape is subject to vagaries of power, influence and finance. Sometimes locally organized groups have been able to access funding to commission artists to create monuments that speak directly of the losses sustained, including the human toll experienced in historic mining disasters which emphasize the sacrifices (Hutchinson, 2019). The welded sculpture produced by local artist Ray Lonsdale, of a miner with his heart torn out, situated in the regenerated Welfare Park in Horden, is an unambiguous cry for past suffering and the conditions that have followed the end of mining. However, contemporary public art mainly offers images of the mining past re-presented as heritage.

The process of transition from mining into new post-industrial conditions was never going to be easy. The memory of the strike adds a layer of particularly fraught emotion, but the UK is not unique in its experience of complex social problems following mine closures. In Canada and across Europe, similar issues have arisen. The particularities of mining, its related organizations, values, and culture have ensured that regeneration is never straightforward. The experience of mining was communal and interdependent; its collective historical memory is easier to incorporate than to displace. Thus, targeted policies of regeneration have attempted to smooth the processes of economic and social change by utilizing surviving natural and cultural resources as a means of safely memorializing the past by mobilizing its relics toward new ends (Wirth et al., 2012). However, and particularly when the closure of mines has been marked by political turbulence, such as in the UK and Nova Scotia in Canada, securing the consent of post-mining populations to new conditions is a delicate task and the process of memorializing is fraught with contested meaning (Haiven, 2010). Troubled memories of past injustices, dangers, disasters and class conflict might be subdued, but they nevertheless continue to haunt the present in ways that have political connotations (Bright and Ivinson, 2019; Spence, 2019). Regeneration decisions intended to smooth transition must find ways to accommodate the past without disturbing or re-igniting the emotions associated with troubles that beset that past.

In the UK, the incorporation of different interests into the post-mining landscape has been achieved partly through a belated commitment to the preservation of remaining buildings, structures, and memorabilia and partly through commissioning new public art that references and memorializes mining. Finance has been made available to energize local populations and encourage them to participate in decision-making about such matters. Notably such participation is focused upon community rebuilding, rather than upon major investment or planning decisions (Atkinson, 1999; Smith and Yellowley, 2019). There has been a largely sympathetic response to locally organized efforts to salvage or replace remnants of the mining industry such as pit wheels and lodge banners. As Scott (2009) has pointed out, these efforts involve a diverse range of people, who having accepted the end of mining, are now keen to acknowledge its role in the history of their local place. The reconstitution of icons is now a function of establishing community identity rather than occupational identity. Even though the politics of mining continue to adhere to the icons, they do so in ways that are mainly celebratory of community cohesion. Whether or not the reworking of these icons can be considered community arts practice, is open to debate. Is a pit wheel an object of art when it has been removed from the mine and resited as a sculptural memory of the mine? Marcel Duchamps' urinal, *Fountain* (1917) comes to mind.

The reconstitution of defunct buildings as galleries and museums, as in Beamish and Woodhorn, is part of a process of reclamation that honors the legacy of mining. Yet, and perhaps as understood by those involved in the orgy of demolition that followed mine closures, it can also serve to highlight the loss. The ex-bank building in which the Mining Art Gallery is housed in Bishop Auckland, is a statement about the value of the art in efforts to rejuvenate a dying place, and at the same time a reminder that the bank no longer thrives in today's Bishop Auckland. That such buildings are available for use as galleries, is symptomatic of the flight of finance and capital that has accompanied de-industrialization over which working class people have had no control. The transformation of Woodhorn Colliery from coal mine to museum with its art gallery housing the Ashington collection is testament to the ways in which history and art focused upon the possibilities of income generation from a volatile post-industrial tourism industry have replaced and appropriated the skeletal remains of reliable, working class employment, and creative leisure (Griffin, 2006). Nevertheless, through the related de-politicized processes of envisioning the post-industrial future whilst memorializing heritage, the reconstitution of mining-related relics and defunct buildings and the controlled creation of new public art can help to ameliorate the disturbed history of transformation.

Buildings that once thrived as part of British industry, can now face a new and brighter future. They are transformed not only with regard to use, but also in relation to context, taking their references from surrounding and related developments as much as from their past. In the case of the Mining Art Gallery, the contextual reference is the ambitious Auckland Project whose intention is to “reinvigorate the region and bring about real change” (Auckland Project, ud.). The Project emerged from the artistic interests of the financier and philanthropist Jonathan Ruffer. In purchasing an important set of Spanish paintings by Francisco de Zurbarát, *Jacob and his Twelve Sons* that hung in the Bishop's palace in Bishop Auckland, Ruffer also took on the vacated palace and its gardens. Ruffer's commitment stimulated an ambitious charitable regeneration venture that has focused upon cultural heritage, linking the long and short histories of the local and the national^8^. The priorities of the Mining Art Gallery can only be understood fully with reference to the greater ambitions for the regeneration of the town which itself did not have a mining history but once benefited from the affluence of the South Durham coalfield and now, hoping to gain income from tourism, links the heritage of the area with a national history.

Highlighting the quality of mining art as high quality “art” in the context of regeneration and the reconstitution of ex-mining places speaks of a will to make a practical contribution to the creation of a brighter future. However, that contribution cannot be made in isolation from broader processes of regeneration and economic redevelopment. In museums and galleries that are costly to establish and run, what counts involves not only judgement about artistic quality, but also potential economic and income-generating potential. Such concerns are integral to the terms of sponsorship, curation, valuation, and advertising that involve the organizational, financial, and personal power of decision-making (Lukes, 2005). Mining art, like any other art, is incorporated into a class-based system of distinction that selects and rejects according to criteria which are not intrinsic to the art itself (Bourdieu and Darbel, 1992). The system of class, money, and power relations that define the terms in which art is valued, excludes the poor and socially discarded (Frascina and Harris, 1992). It includes neither Jimmy Kays nor the populations of ex-mining places. What is promoted for public consumption depends upon who has decision-making power in a marketing context that includes selling exhibitions and the goods associated with them to as wide an audience as possible. In these terms, the appeal to national nostalgia and sentiment is inextricably connected with the emphasis on the quality of the art.

## Artistic Quality

**Figure 6:**
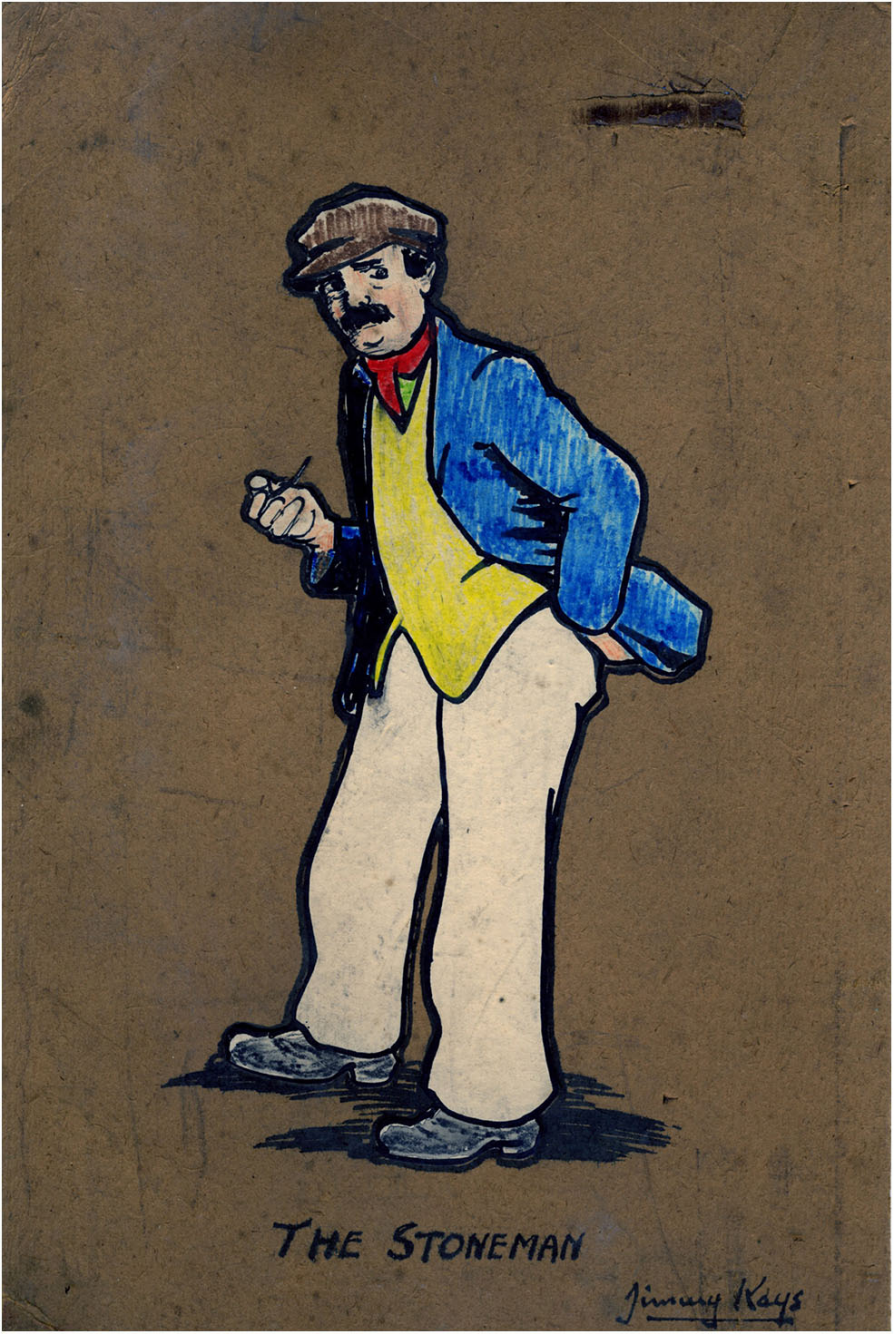
The Stoneman. *Author's collection*.

**Figure 7:**
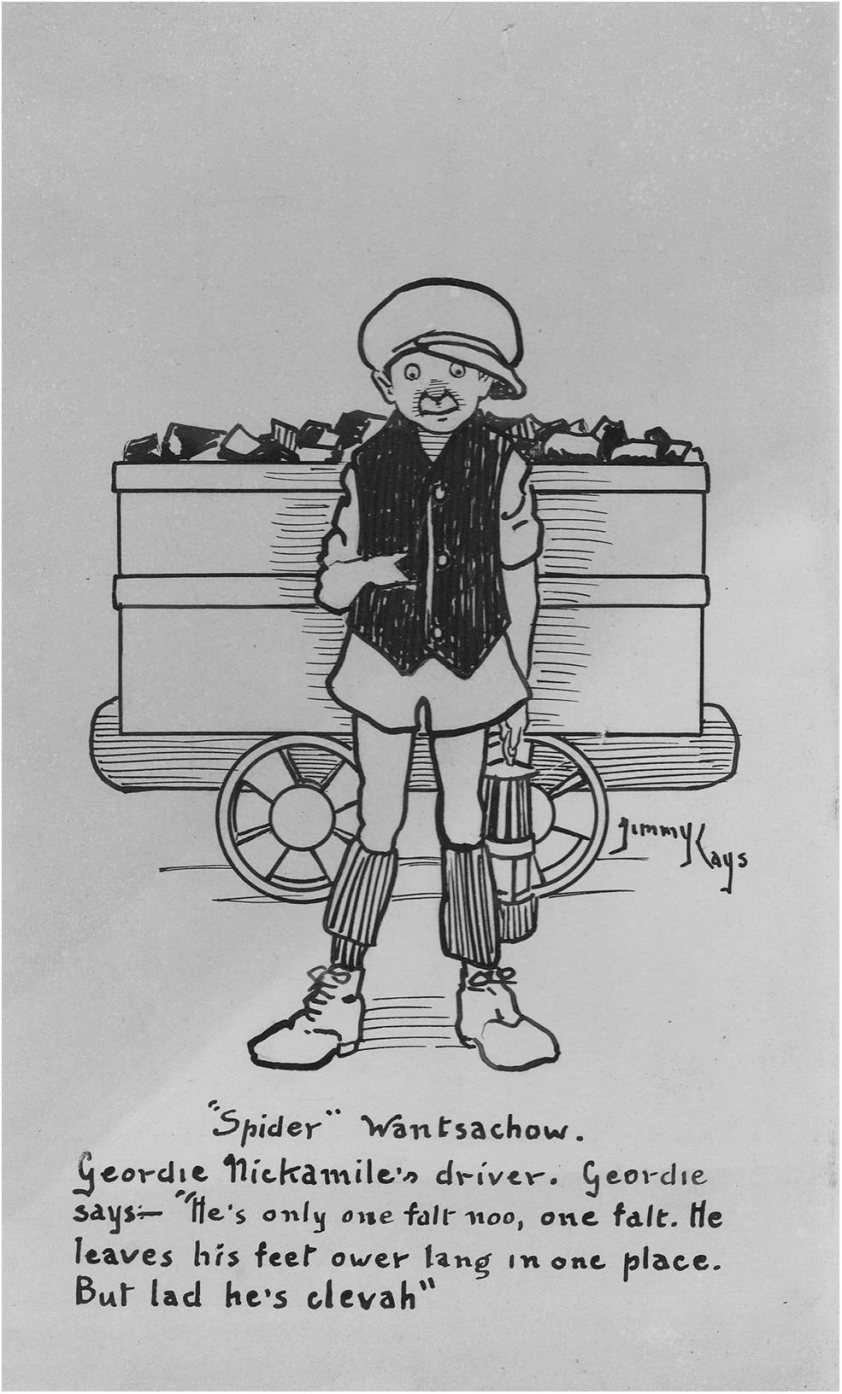
“Spider” Wantsachow. *Private collection R. Gibson*.

**Figure 8:**
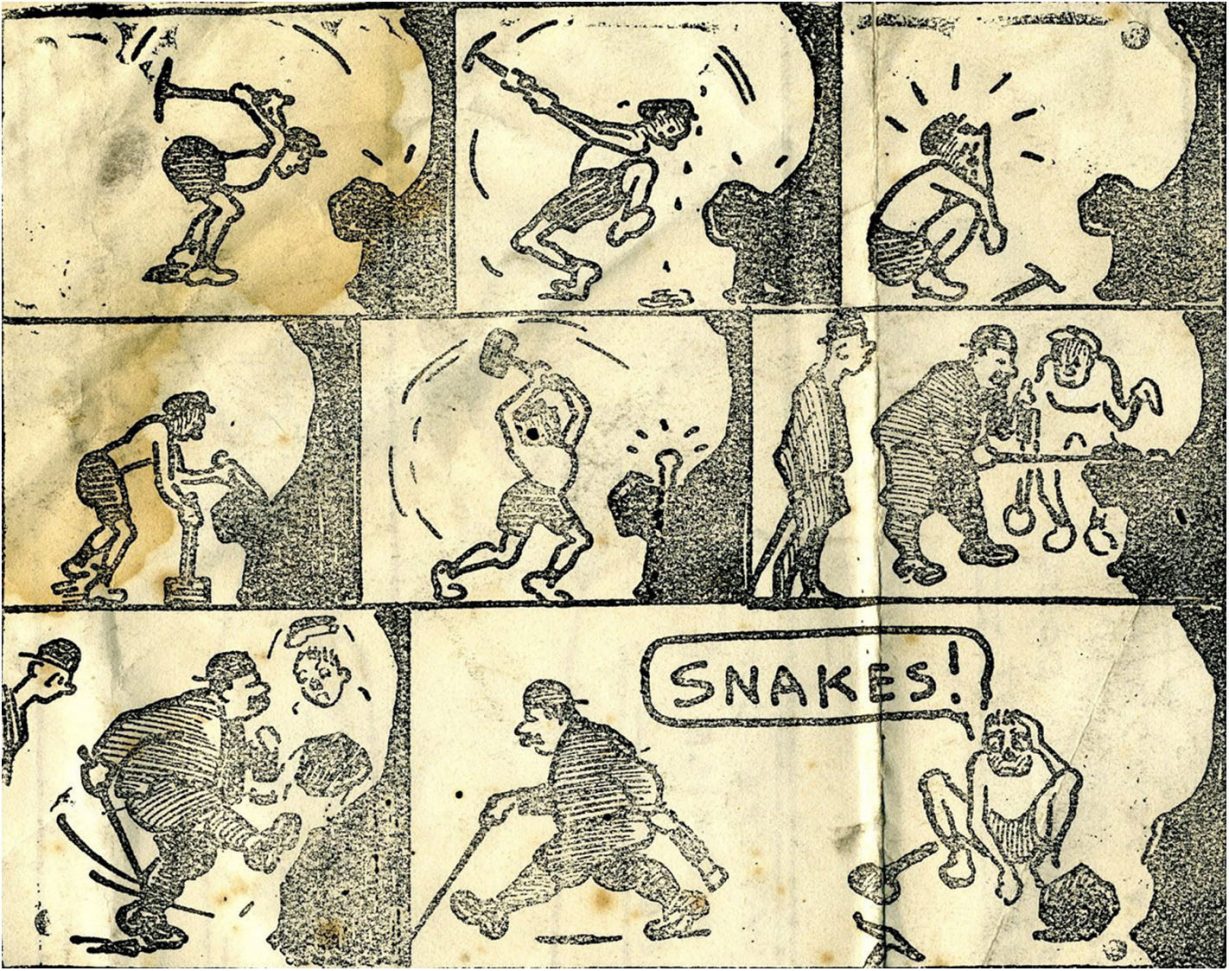
Snakes! *Unattributed newspaper cutting, author's collection*.

**Figure 9:**
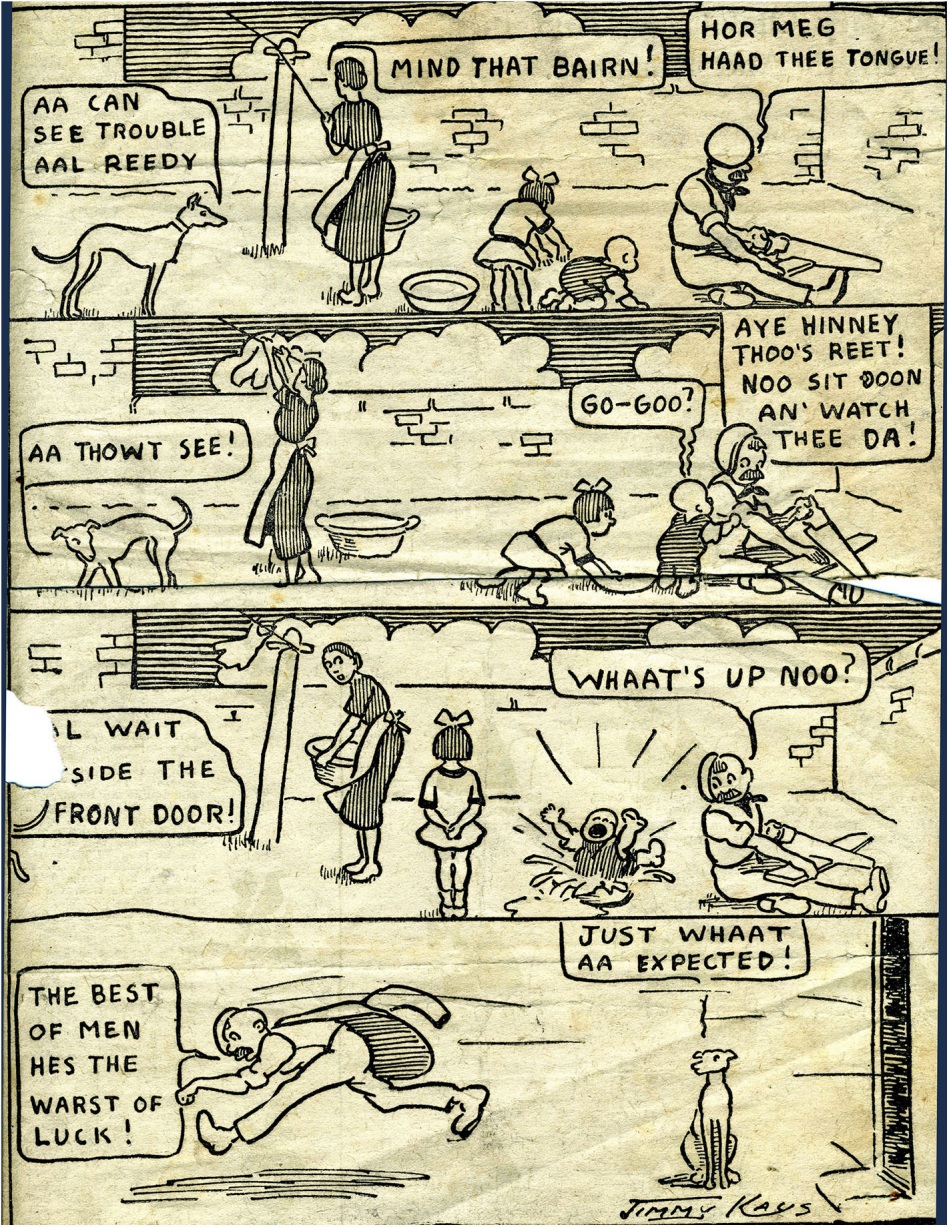
The warst of luck! *Unattributed newspaper cutting, author's collection*.

**Figure 10:**
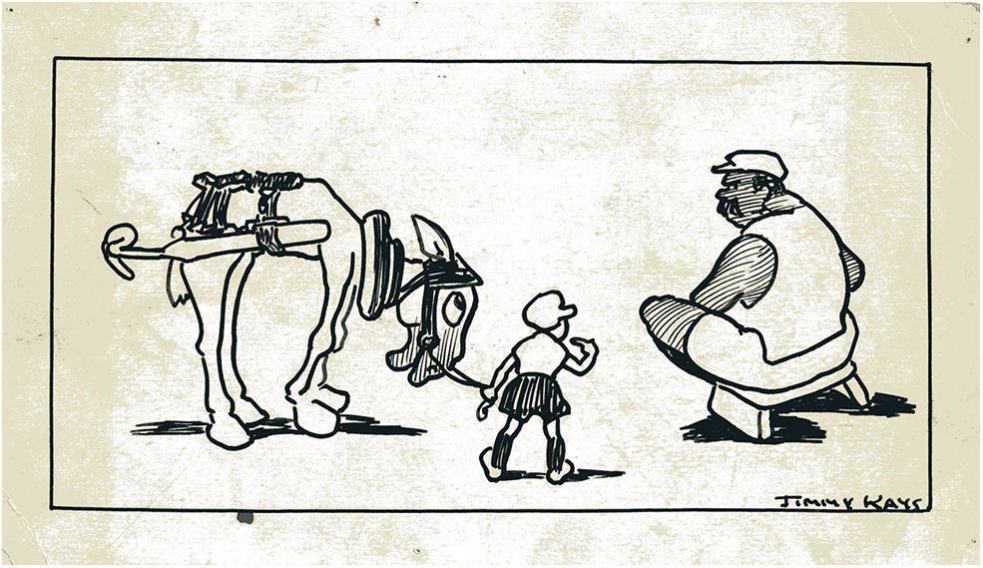
Hewer, putter and pit pony. *Author's collection*.

**Figure 11:**
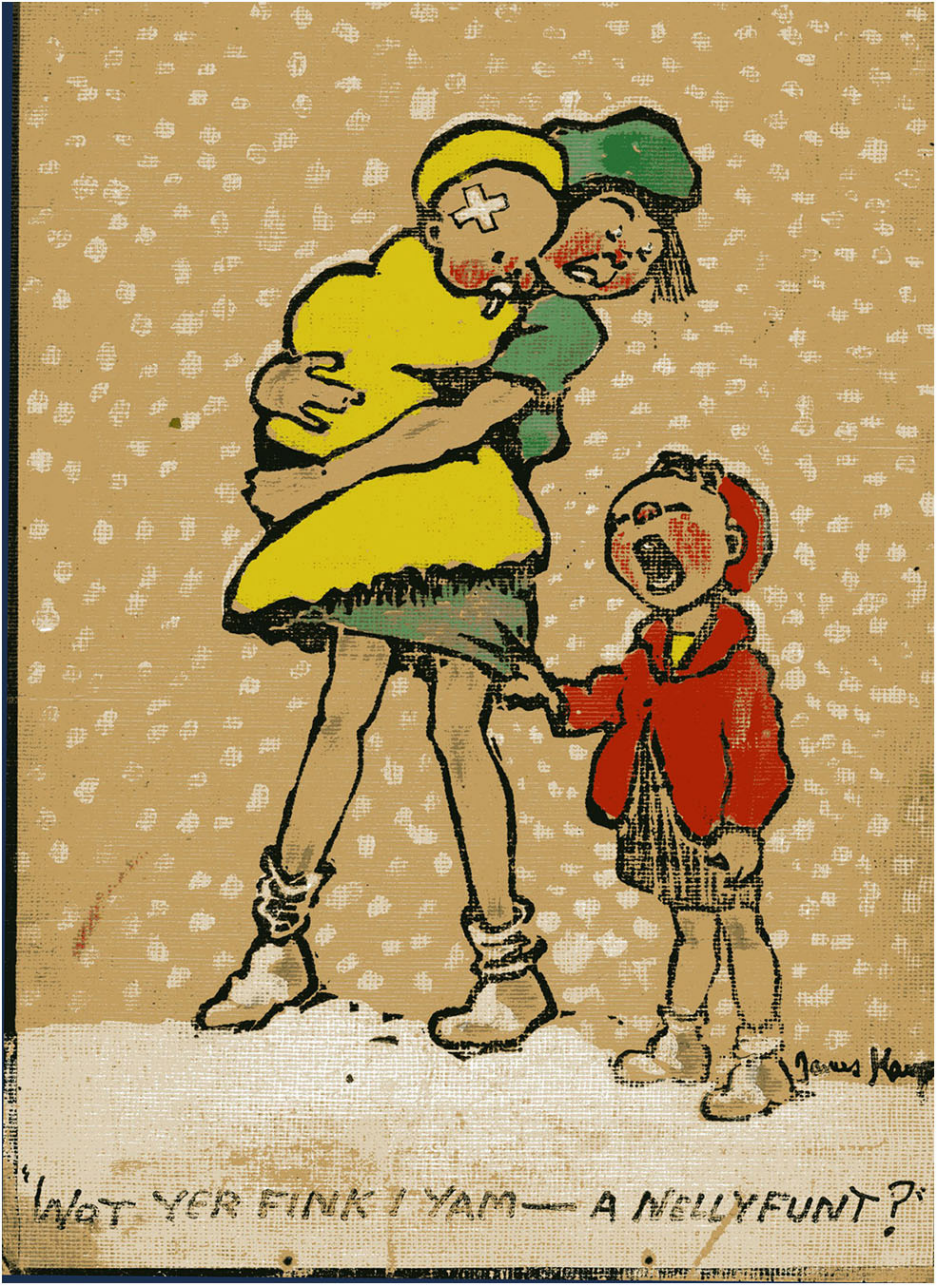
A Nellyfunt? *Private collection, C. Kays*.

The art of the miner artists demonstrates talent and skill but the skills differ from those demonstrated by Jimmy Kays who was interested in character and relationships rather than the drama of mining or the everyday patterns of mining life. Rather than fully realized paintings, he used outline portraits and humor to emphasize qualities of character (Figures 6, 7), and cartoons to illustrate what are mainly dislocated and uneven relationships (Figure 8). He shows working hierarchies and camaraderie in the mine. The struggle with the coal face, and the dangers are present, but the focus is elsewhere—with the communication between underground workers. Above ground he shows miners discomforted by misunderstanding in relationships with women (Figure 9), with other authorities such as police, and in public institutions such as theaters. In illustrating the world of children, he is alert to the nature of their friendships, their interests and their trials and shows particular sympathy for young boys attempting to adapt to underground work (Figure 10), and girls laden with the responsibilities of childcare (Figure 11).

Kays dependence on the cartoon form mean that he made little use of the light and shade effects, the chiaroscuro, that gives weight and volume to the underground images of working miners, whilst emphasizing the atmosphere of industrial energy and power in the overground mine images. Many of the paintings by the most highly rated artists including those still living, that specifically depict miners and mines, feature strong light contrasts, and use a limited color palette.

The particular use of chiaroscuro in mining art not only represents the reality of the darkness punctuated by artificial light in underground conditions, but is also part of an industrial aesthetic in which coal mining represents the struggle of man against nature that is symptomatic of modern progress and, particularly in the case of McGuinness, has religious allusions (McManners and Wales, 2006). Dualistic metaphors focusing upon day and night, heaven and hell, spiritual and physical, good and evil are persistent features of coal mining art (Thesing, 2000). Within this iconography, the miner appears as an heroic figure, pitting his body against the unyielding and unforgiving forces of nature. This is a post-enlightenment narrative of the industrial revolution and integral to the national story of the UK as a world power. In the dominant visual narrative, the struggle of miners as a body of workers against exploitation by mine owners and subject to the vagaries of the market for coal, is subsumed by the greater struggle against forces of nature and of darkness.

Mining paintings depicting the coal face and the pit head are often at one and the same time realistic and idealistic, literal and symbolic. The battle against nature presents the miner as industrial warrior. Implicit in this is the notion of miner as national and working class hero. Until the 1984 strike, in the labourist politics pursued within mining, there was no apparent contradiction between national and class heroism: miners' trade unionism and the history of struggle was for better conditions and wages, not for revolution.

Kays images do not show the miner as any sort of hero. “The Kist” is the only Kays drawing that includes underground light and shadow effects, but there is no drama in the scene, and no particular attention to the masculine physicality of the miners. His other mining images suggest the wearying everyday attrition of mining rather than the more spectacular dangers associated with roof falls and explosions. His sensibility, especially with the reproduced images of the broken props holding the roof (see Figure 4), is anxiety for what might happen. The audience for the published cartoons, readers of the *Weekly Star*, belonged to the same class and the same locality as Jimmy Kays. Otherwise his work was only for himself and kith and kin. Using an easily understood art form, he was speaking to the people whose worlds and worries he shared. There is no heroism in the humor, but rather what is revealed is a grim resilience and vulnerability in the face of forces and circumstances beyond the control of the laboring miner.

The cartoons employ physicality to emphasize informal relations of power in the pit, rather than “man against nature.” The oppressed boy miner and pit pony, the ineffectual deputy, and the bullying coal hewer express realities of underground relations that do not conform to the heroics of mining (see Figure 10).

To survive conditions that constantly threaten to overwhelm, the miners in the narrative developed by Kays, including the boys and ponies for whom he shows particular sympathy are often hapless. They are mainly resigned to their conditions and appear powerless in the face of forces that are beyond their control. Their only resource is their wry humor, their companionship and their own characters. This is no heroism. It is simply endurance and it does not lend itself to a sentimental or romantic reading.

Kays' depiction of life above ground is interesting in the way it depicts male and female worlds as full of misunderstanding (see Figure 9). The sexual division of labor in mining ensured that the home was the domain of women but unlike other miner artists who tend to show co-operation and tenderness in the home, Kays shows men and women misunderstanding each other, and alienation of the miner from the domestic situation.

The retreat for the miner above ground is in male company and sometimes in alcohol. It is impossible to take from Kays' images any sense of glory in mining. That does not make it irrelevant as mining art, but it does problematize its position. It sits outside the mining art narrative and does not conform to the heritage tropes that make mining art attractive to the leisure and tourist markets. Yet it offers a unique visual historical record of its time and one with which local people in Durham identify and understand.

When the Jimmy Kays pictures, including some from the Kays family collections, were copied, printed, framed, and exhibited in local venues in East Durham, they attracted large audiences. Apart from the Art Block and Horden Heritage Center, Kays prints were shown at Horden Centennial Center, Blackhall Community Center and Seaton Holme, Easington. The physical spaces available in these community settings included corridors, rooms where no nails were allowed in the walls, and rooms that were outside the main congregating areas of the building. Nevertheless, these venues have a present relevance in local lives, and as such, the decision to display the Kays work in them was to signify that this art work was meant to be viewed by those who use the buildings now, albeit mainly for other purposes. In addition, the work has been shown on display boards and tables in conference and festival settings, such as the Easington Miners' Picnic in 2019.

The authenticity of Kays as a local miner artist was important in the promotion of the exhibitions which were intended to be accessible to and to communicate with an audience of people living in post-mining circumstances. Nobody who visited these exhibitions questioned the quality of the art, no market relationships were involved, and the audience engaged with the images as equals rather than as consumers. That “one of their own” had been “discovered” and was being recognized by having his work shown in an exhibition was reason itself for pride, but what was most important was the affirmation of class and cultural identity that communicated directly to deep knowledge and common understanding without any intervening “arts” discourse and without any question about how much the art might be “worth.” Local visitors were excited by the representation of a history that they recognized as uniquely their own, by images of an everyday life which otherwise remained only in traces, detritus, and the mementos hidden in family cupboards. Some simply looked quietly and reflectively. Others were stimulated to speak. Their conversations revealed a poignancy about mining always touched by ambiguity, sad for loss but rarely nostalgic. The examples of responses that I am able to give depend entirely upon my own presence at accidental moments and my ability to recollect what was said. Three examples which are uppermost in my recollection will serve to illustrate the significance of Kays' work and its relevance as working class art.

**Figure 12:**
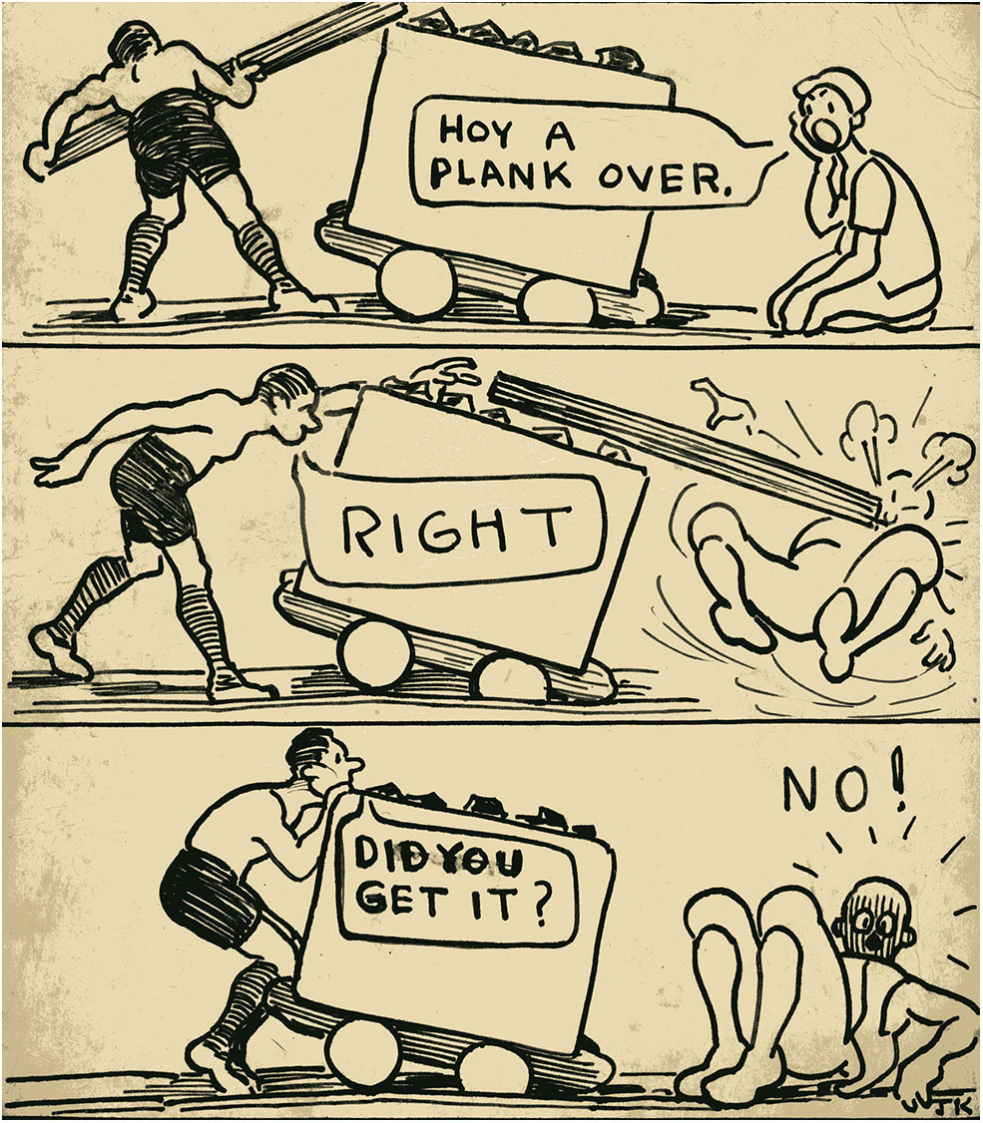
Hoy a plank over. *Author's collection*.

The first example is that of a cartoon whose humor belies its seriousness (Figure 12).

This cartoon, illustrating everyday dangers in the pit, frequently provoked a response. One ex-miner explained that the first underground job that he had been given was to lift a truck back onto the rails. Another described the narrowness of the tunnels and the fact that often there was no space at the sides to take the plank around and so it had to be thrown (hoyed) over^9^. In both these cases, the men wanted very much to speak of “what it was like.” Meanwhile an elderly woman wanted to tell of her grandfather. She said that he had been a very strong man; it was told that he was so strong that he could lift a tub back on the rails on his own, with his bare hands, without a plank. Before he died, he was like a matchstick and could have been knocked over by a child. His decline and death was due to pneumoconiosis, the miners' lung disease caused by inhaling coal dust over years of underground toil. This is a well-known mining disease, but not one that conjures images of heroism. That it does not appear as such in Kays' images does not invalidate the fact that his cartoon provoked this memory and brought the topic into public conversation through a private story. Unlike the mines themselves, pneumoconiosis has not yet disappeared. It is a hidden legacy of mining that continues to blight the lives of aging miners—including incidentally, an ex-Horden miner who is a member of edan and who uses art to give himself a life beyond his disability.

The second example concerns a retired woman, a volunteer in the Horden Heritage Center. For her, the fact that these were cartoons was of particular significance, mainly because of their immediacy and because of the dialect captions. She was delighted by the humor announcing that it was “ages” since she had laughed like she did when viewing these cartoons. They had apparently resurrected an aspect of her being that had been repressed and silenced with the loss of the mines and their associated speech patterns. She was not nostalgic for this lost world, but it connected with her efforts in the Heritage Center to offer a community café and to her role in collecting and organizing local memorabilia, documents, and photographs to be accessed there. Kays' cartoons spoke in a voice she knew, offering encouragement for her recording and archiving work and feeding into the conversations in the center. In the Heritage Center the Kays exhibitions were a reminder that local talent and agency had survived the depredations of mining. They might now inspire local confidence to survive the dire conditions of the present, where in 2015, an ex-mining house, including that in which Kays parents lived, could be purchased for £1.00 (Jenkins, 2015). Jimmy Kays might have belonged to a different age, but the spirit of fortitude and the subtleties of humor deployed as a social defense against adversity remain relevant.

**Figure 13:**
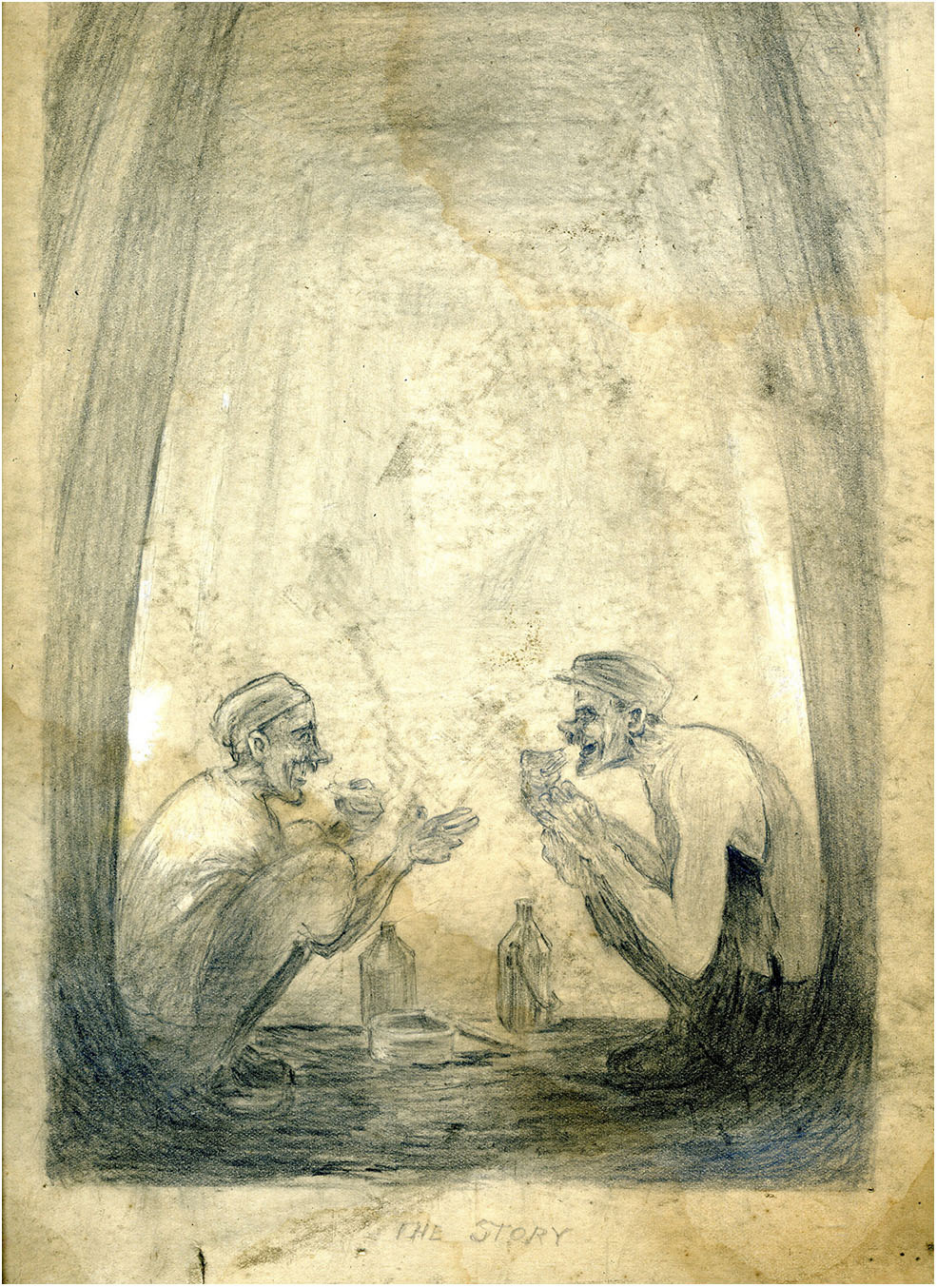
The story. *Author's collection*.

The thwarted ambitions inscribed in Kays' life and work speak to the post-mining present. He was not the only miner for whom pit work was a necessity preventing the fulfillment of other talents. One Art Block diary entry records that an ex-miner who visited the Kays exhibition said that “many miners were very good drawers” (Art Block, 2016). Another was moved to bring some pen and ink drawings of underground scenes that he had done himself. The third example concerns an ex-miner from Horden who helped to hang the Kays exhibition in the Heritage Center. He was reminded of the art in underground tunnels, mentioning chalked cartoons and murals that workers had done themselves. Subsequently, other ex-miners have corroborated this. It appears that drawing underground was not uncommon. Some of it was lewd—understandably perhaps, but some seems to have been a commentary on the work itself. It is recorded that it was the result of seeing Tom McGuinness drawing on a coal tub that prompted a mining supervisor to suggest that he should attend evening art classes (Borchard, ud.). Underground art created by miners was lost with the mine but some sketches using conventional art materials, that were done *in situ* survive. There is at least one image in the Kays collection that might have been drawn underground. This sketch entitled “The Story” shows two miners eating their bait and engaged in conversation (Figure 13). Here the context is secondary to the talk; for the men, the pleasure of “the story” is a distraction from their surroundings. This faded pencil drawing shows something of the importance of conviviality in underground relationships.

Creativity in the squalid and inhospitable conditions of work underground, prompts questions about the exclusion of opportunities for creativity in contemporary workplaces. As a new member of edan, recently moved to County Durham asked, “What would be the equivalent today of mining art?” Indeed, what would be the equivalent? Is it simply that contemporary work lacks the visual qualities, the dramatic lighting, and the icons that inspired mining art? Or is it that surveillance and methods of controlling workers in post-industrial conditions excludes all opportunity for independent creativity? Today, “cultural production” has become part of a contrived response to degraded localities. In the neo-liberal economy and workplace, creativity is only legitimized if harnessed for the benefit of productivity: artistic practice has become instrumental (Mould, 2018). Creative production is defined as “art” only in these terms. There are few remaining circumstances in which workers, including professional artists, can feel that their creativity is legitimate or has value outside the market. Moreover, representing “what you know” in a social sense hardly seems meaningful in local societies characterized by fragmentation, polarization, and mourning for the past. Disparate, tightly controlled working conditions and privatized and individualized leisure militate against opportunities for producing visual art that can be collectively representative of contemporary life and culture. More than ever, art is removed from its social bearings, dislocated from everyday life and presented as unattainable for ordinary people—no matter what their talent. The challenge arising from the world of Jimmy Kays is to consider how artistic practice in post-mining localities might be reoriented to the present.

## Conclusion

The strength of the art work produced by Jimmy Kays is also its weakness. People who have experienced and inherited the consequences of mine closures, whose creativity has been crushed and appropriated through their displacement, find in Kays' images features that connect them positively with their past without provoking a desire for its return. Kays' drawings and cartoons, produced without reference to a consuming audience, are inherently valuable in terms of the historical insight they offer into County Durham mining life in the first quarter of the twentieth century. Yet its unmediated authenticity also renders Kays' work problematic in the contemporary conditions in which mining art is flourishing. It sits uneasily in the neo-liberal post-industrial conditions in which mining art is accorded value and brought into public recognition. It is physically shabby. It threatens the cohesive narrative of a “hard” but heroic and glorious remembered past through which so much mining art is viewed and filtered, and provokes an encounter with a longer history of daily attrition and denigration in which miners and their families struggled to create a distinctive, resilient culture.

The class conflicts between miners and mine owners before nationalization, and subsequently between miners and the state are incorporated in most mining art by their relegation to a past which seems to have no implications for the present. This appeals to a critical but ultimately passive nostalgia for a period from which emerged a better present. Such a conservative reading situates the class-based mining conflicts in that same past. The national story is one which transcends such conflict. It is impossible to view the work of Kays in these terms because his art was of a moment of abjection—more continuous than discontinuous with post-mining conditions. It was local, immediate, and unsentimental, produced without symbolism or reference to a wider set of sensibilities about “art.” This does not make it “not art.” The power to define the worth of art is not a power without borders, but is circumscribed by a range of interest groups, including those who deal in art on the open market, those who influence decisions in galleries, museums, art journals, art education, and universities, each of which have their own markets to satisfy and all of whom are interconnected.

The terms of the historical invisibility of Jimmy Kays, the production of his work in conditions of poverty and without professional patronage other than his brief period cartooning for a short-lived working class newspaper, the preservation of his work in a private domestic setting, and the difficulties of including it in the public lexicon and display of “mining art,” illuminates the exercise of class-based hegemonic power in terms of the production, consumption and range of meaning inscribed within all art. Kays possessed artistic skill, but he is a minor artist whose output is outside market valuation except as a historic curiosity. It is a unique visual “insider” representation of a time and place which is otherwise largely unseen and forgotten but it could not reveal as much as it does without the skill of the artist. Its intrinsic value is that it speaks for and to those who have been largely unseen and unheard, whose lives are not reached by filtered and mediated historical narratives.

Not all people can be artists, but all people have creative potential. It is the expression of creativity that facilitates human confidence and agency. Restoring that confidence and agency amongst people discarded by the death of mining and denigrated in the post-mining world cannot be undertaken by art alone, and art cannot replace a world that is lost. However, mining art can make a contribution. Displayed in appropriate and accessible conditions, it can facilitate remembrance, reflection, and conversation in ways that help to inspire pride and confidence in identities that otherwise have few unmediated outlets. To fulfill this potential requires that a widening of the scope of understanding about what is valuable in art, taking into consideration the class based criteria that promote or denigrate different forms of creative expression. We need to foster a creativity and artistic practice that is integral to existence, questioning of prevailing conditions, and empowering for those engaged in it. Mining art can support this, but only through a more critical interrogation that also recognizes that in class terms a minor artist in one setting, can be a major artist in another.

## Author Contributions

The author confirms being the sole contributor of this work and has approved it for publication.

## Conflict of Interest

The author declares that the research was conducted in the absence of any commercial or financial relationships that could be construed as a potential conflict of interest.

## Footnotes

^1^This information was offered by his surviving son, Colin Kays.

^2^Durham Heritage Coast is a multi-agency partnership organization that focuses upon protecting and enhancing the qualities of the coastal zone which is also the area of Durham's coastal collieries–the last in the Durham coalfield to close.

^3^“Pitmatic” was the language used by Durham miners to communicate amongst themselves.

^4^Much of this material is now available in archives held by organizations such as the British Film Institute (www.bfi.org.uk), the National Coal Mining Museum (www.ncm.org.uk), and the North of England Institute of Mining and Mechanical Engineers (www.mining institute.org.uk).

^5^See for example the commissioned work on show at Bowes museum in 2019 as part of the centenary of his birth. That work is competent but lacks much of the energy of his earlier images of mining life.

^6^In the first stage of Creative People and Places in East Durham, professional organizers stated that there would be no references to mining in their work. This stance was unpopular locally and was changed after evaluation.

^7^A “kist” was an underground cell where the miners met before approaching and after leaving the coal face. In this cell, the deputy (overman), distributed tasks and work tools.

^8^It includes the highly rated tourist attraction, “Kynren” that relates a 2,000 year history of England in an outdoor theatrical show.

^9^One of the few known miner artists from the early years of the twentieth century, Gilbert Daykin shows the narrowness of the tunnels in a fully realized oil painting, “The Tub: At the end of the coalface” (1934).

## References

[B1] Art Block (2016). Diary Entry. Seaham: Edan.

[B2] Arts Council (2017). Creative People and Places 2020-24: Round 2. Available online at: https://www.artscouncil.org.uk/get-funding/creative-people-and-places (accessed June 26, 2020).

[B3] Atkinson R. (1999). Discourses of partnership and empowerment in contemporary urban regeneration. Urban Stud. 16, 59–72. 10.1080/0042098993736

[B4] Auckland Project (ud.). Our History. Available online at: https://www.aucklandproject.org/about/ourhistory/ (accessed June 26, 2020).

[B5] Baccaro L. Howell C. (2011). A common neoliberal trajectory: the transformation of industrial relations in advanced capitalism. Polit. Soc. 39, 521–563. 10.1177/0032329211420082

[B6] Beatty C. Fothergill S. Gore T. (2019). The State of the Coalfields 2019: Economic and Social Conditions in the Former Coalfields of England, Scotland and Wales. Report commissioned by the Coalfields Regeneration Trust, Sheffield Hallam University. 10.7190/cresr.2019.6676686343

[B7] Bennet K. Beynon H. Hudson R. (2000). Coalfields Regeneration: Dealing With the Consequences of Industrial Decline. Bristol: Policy Press for JRF.

[B8] Borchard R. (ud.). The Ruth Borchard Collection: Tom McGuinness. Available online at: https://www.ruthborchard.org.uk/collection/tom-mcguinness/ (accessed March 20, 2020).

[B9] Bourdieu P. Darbel A. (1992). The Love of Art, in Art in Modern Culture: An Anthology of Critical Texts, eds Frascina F. Harris J. (London: Phaidon Press Ltd.), 174–180.

[B10] Boym S. (2001). The Future of Nostalgia. New York, NY: Basic Books.

[B11] Boym S. (2015). The future of nostalgia. Shifter Magazine. Available online at: https://shifter-magazine.com/wp-content/uploads/2015/11/Bpym-Future-of-Nostalgia.pdf (accessed June 9, 2020).

[B12] Bright G. Ivinson G. (2019). Washing lines, whinberries and reworking “waste ground”: women's affective practices and a haunting within the haunting of the UK coalfields. J. Work. Class Stud. 4, 25–39. Available online at: https://workingclassstudiesjournal.files.wordpress.com/2019/12/jwcs-vol-4-issue-2-dec-2019-bright-ivinson.pdf

[B13] Castlegate House (2019). Norman Cornish. Available online at: https://www.castlegatehouse.co.uk/paintings-for-sale/norman-cornish (accessed March 9, 2020).

[B14] Davis R. L. Cousins J. (1975). The “New working class” and the old, in Working-Class Images of Society, ed Bulmer M. (London: RKP), 192–205.

[B15] Dowson J. (2019). Income, Salaries and Wages, Northumberland 2019. Northumberland Knowledge Research Report, Northumberland County Council. Available online at: www.northumberland.gov.uk/Northumberland-knowledge-and-JSNA.aspx (accessed June 9, 2020).

[B16] Engelbrecht G. (2015). Recovered work of artist showcased. Northern Echo, p. 7.

[B17] Feaver W. (1988). Pitmen Painters: The Ashington Group 1934–1984, London: Chatto and Windus.

[B18] Frascina F. Harris J. (eds.). (1992). Introduction, in Art in Modern Culture: An Anthology of Critical Texts (London: Phaidon), 10–15.

[B19] Freeth H. A. (1951, December). Joseph rushton of bank hall colliery, Pit Profile No. 56, *Coal: The Magazine of the Mining Industry* 9.

[B20] Garside W. R. (1971). The Durham Miners 1919–1969. London: George Allen and Unwin.

[B21] Gilchrist R. Jeffs T. (2001). Settlements, Social Change and Community Action. London: Jessica Kingsley.

[B22] Goodman G. (1985). The Miners' Strike. London: Pluto Press.

[B23] Gordon A. F. (2008). Ghostly Matters: Haunting and the Sociological Imagination. Minneapolis, MN: University of Minnesota Press.

[B24] Griffin C. (2006). ‘Instead of manufacturing goods, we are manufacturing heritage’: the national coalmining museum for England. Labour Hist. Rev. 71, 289–302. 10.1179/174581806X164595

[B25] Griffiths B. (ed.). (1994). Durham and Around: A Dialect Reader. Seaham: Amra Imprint.

[B26] Haiven L. (2010). Regeneration among coal-mining communities in Canada and the UK. The role of culture, in Interrogating the New Economy: Restructuring Work in the 21st Century, eds Pupo N. Thomas M. (Toronto, ON: Broadview), 195–213.

[B27] Hall L. (2008). The Pitmen Painters. London: Faber and Faber.

[B28] Hall L. (2009). Introduction, in BFI Booklet Accompanying DVD, Portrait of a Miner: National Coal Board Collection, Vol. 1 (London: British Film Institute).

[B29] Hall T. (1981). King Coal: Miners, Coal and Britain's Industrial Future. Harmondsworth: Penguin.

[B30] Hannington W. (1937). The Problem of the Distressed Areas. London: Gollancz.

[B31] Hansard (2018). Arts Council England Funding: Coalfield Communities, Vol. 636. Available online at: www.hansard.parliament.uk (accessed June 24, 2020).

[B32] Henderson T. (2015). Late Lawer's Pitmen Painter Collection Goes up For Auction in Newcastle, Chronicle. Available online at: www.chroniclelive.co.uk (accessed June 26, 2020).

[B33] Horton H. (2019, November 14). Art world pays homage to “The rembrandt of the coal mines.” Daily Telegraph, p. 10.

[B34] Hutchinson L. (2019). A Sneak Preview of the Amazing Statue Dedicated to Langley Park Miners More Than 40 Years After Pit Closures. Report in the Chronicle. Available online at: www.chroniclelive.co.u.k (accessed February 9, 2020).

[B35] Illingworth L. G. (1952). Shortsighted. Cartoon. Punch, p. 79.

[B36] Jenkins C. (2015). ‘It's Like Beirut’: The Town Where Homes Are on Sale for £1, Channel 4. Available online at: https://www.channel4.com/news/horden-county-durham-bedroom-tax-one-pound-housing (accessed March 20, 2020).

[B37] Ling C. Handley J. Rodwell J. (2007). Restructuring the post-industrial landscape: a multifunctional approach. Landscape Res. 32, 285–309. 10.1080/01426390701318171

[B38] Lukes S. (2005). Power: A Radical View. London: Palgrave Macmillan. 10.1007/978-0-230-80257-5

[B39] McManners R. Wales G. (2002). Shafts of Light: Mining Art in the Great Northern Coalfield. Durham: Gemini Productions, Co.

[B40] McManners R. Wales G. (2006). McGuinness: The Art of Tom McGuinness. Durham: Gemini Productions, Co.

[B41] McManners R. Wales G. (2008). Way to the Better: The Spennymoor Settlement. Durham: Gemini Productions, Co.

[B42] Mould O. (2018). Against Creativity. London: Verso.

[B43] Munday M. (2017). Working With Social Haunting: Seaham. Available online at: https://www.socialhaunting.com/blog/seaham (accessed June 9, 2020).

[B44] Norman Cornish Ltd. (2019). Available online at: https://www.normancornish.com (accessed June 24, 2020).

[B45] Perchard A. (2013). Broken men” and “Thatcher's children”: memory and legacy in the Scottish coalfields. Int. Labour Work. Class Hist. 84, 78–98. 10.1017/S0147547913000252

[B46] Popple S. MacDonald I. W. (eds.). (2012). Digging the Seam: Popular Cultures of the 1984/5 Miners' Strike. Newcastle upon Tyne: Cambridge Scholars.

[B47] Prince D. (2015, June 13). Family's pride as pitman's art goes on show. Sunderland Echo. p. 8–9.

[B48] Roberts I. (2007). Collective representations, divided memory and patterns of paradox: mining and shipbuilding. Sociol. Res. Online 12, 1–19. 10.5153/sro.1611

[B49] Samuel R. Bloomfield B. Boanas G. (1986). The Enemy Within: Pit Villages and the Miners' Strike of 1984–5. London: RKP.

[B50] Scott C. (2009). Contemporary expressions of coal mining heritage in the Durham coalfield: the creation of new identities. Folk Life 47, 66–75. 10.1179/175967009X422774

[B51] Siddall N. (1980). ‘Foreword’ in Coal: Mining in British Art, 1680–1980. Catalogue of an Exhibition Organised by the Arts Council of Great Britain With the National Coal Board and Supported by Barclays Bank. London: Arts Council.

[B52] Smith K. Yellowley T. (2019). Echoes of the North East Miners: Some Last Traces of the Collieries and Tributes to the Pitmen. Newcastle-on-Tyne: Tyne Bridge Publishing.

[B53] Sönnichsen N. (2020). Number of People Employed in the Coal Mining Industry in the United Kingdom (UK) From 1920 to 2018 (in 1,000s). Available online at: https://www.statistica.com/statistics/371069/employment-in-coal-mining-industry-in-the-united-kingdom-uk/ (accessed June 20, 2020).

[B54] Spence J. (1996). Women, wives and the campaign against pit closures in county Durham: understanding the vane tempest vigil. Fem. Rev. 60, 33–60. 10.1080/014177898339389

[B55] Spence J. (2015). The Lost World of Jimmy Kays. Seaham: Edan.

[B56] Spence J. (2019). Twisted seams: a gendered social haunting. J. Work. Class Stud. 4, 5–24. Available online at: https://workingclassstudiesjournal.files.wordpress.com/2019/12/jwcs-vol-4-issue-2-dec-2019-spence.pdf

[B57] Spence J. Stephenson C. (2007). Female involvement in the miners' strike: trajectories of activism. Sociol. Res. Online 12, 1–11. 10.5153/sro.1461

[B58] Spence J. Stephenson C. (2009). Side by side with our men?” Women's activism, community and gender in the 1984–1985 British miners' strike. Int. Labour Work. Class Hist. 75, 68–84. 10.1017/S0147547909000064

[B59] Spence J. Stephenson C. (2012). Women's poetry and the politics of the personal in the 1984–85 miners' strike, in Digging the Seam: Popular Cultures of the 1984/5 Miners' Strike, eds Popple S. MacDonald I. W. (Newcastle upon Tyne: Cambridge Scholars Publishing), 153–169.

[B60] Spennymoor AAP (2017). Statistical Profile, Research and consultation Team, Durham County Council. Available online at: https://www.durhaminsight.info/spennymoor-aap/ (accessed June 26, 2020).

[B61] Stoner S. (2015, January 27). A snapshot of life in the pits. Sunderland Echo, p. 34–35.

[B62] Strangleman T. (2013). Smokestack nostalgia,” “Ruin porn” or working class obituary: the role and meaning of deindustrial representation. Int. Labor Work. Class Hist. 84, 23–37. 10.1017/S0147547913000239

[B63] Thesing W. B. (ed.). (2000) Caverns of the Night: Coal Mines in Art, Literature and Film. Columbia, SC: University of South Carolina Press.

[B64] Williams G. (ed.). (2009). Shafted: The Media, the Miners' Strike and the Aftermath. London: CPBF.

[B65] Williams G. (ed.). (2019). Shafted, 2nd Edn. Newcastle upon Tyne: CPBF North.

[B66] Williamson B. (1982). Class, Culture and Community: A Biographical Study of Social Change in Mining. London: RKP.

[B67] Wirth P. Cernič-Mali B. Fischer W. (eds.). (2012). Post-Mining Regions in Central Europe: Problems, Potentials, Possibilities. Munich: OEKOM.

[B68] Young J. (2007). The Vertigo of Late Modernity. London: Sage.

